# Heterointerface‐Engineered SiC@SiO_2_@C Nanofibers for Simultaneous Microwave Absorption and Corrosion Resistance

**DOI:** 10.1002/advs.202509071

**Published:** 2025-07-31

**Authors:** Limeng Song, Feiyue Hu, Yongqiang Chen, Li Guan, Peigen Zhang, Linan Wang, ZhengMing Sun, Yanqiu Zhu, Hailong Wang, Renchao Che, Bingbing Fan, Rui Zhang

**Affiliations:** ^1^ Henan Key Laboratory of Aeronautical Materials and Application Technology, Henan International Joint Laboratory of Aeronautical Function Materials and Advanced Processing Technology, School of Material Science and Engineering Zhengzhou University of Aeronautics Zhengzhou 450015 P. R. China; ^2^ State Key Laboratory of Engineering Materials for Major Infrastructure School of Materials Science and Engineering Southeast University Nanjing 211189 P. R. China; ^3^ School of Materials Science and Engineering Zhengzhou University Zhengzhou 450001 P. R. China; ^4^ Institute of Advanced Ceramics Henan Academy of Sciences Zhengzhou 450046 P. R. China; ^5^ Department of Engineering Faculty of Environment Science and Economy University of Exeter Exeter EX4 4QF United Kingdom; ^6^ Laboratory of Advanced Materials Shanghai Key Lab of Molecular Catalysis and Innovative Materials Academy for Engineering & Technology Fudan University Shanghai 200438 P. R. China

**Keywords:** corrosion resistance, heterointerface engineering, microwave absorption, multifunctional coatings, SiC@SiO_2_@C nanofiber

## Abstract

To meet the demands of maritime defense and transportation, next‐generation microwave absorption (MA) materials must combine efficient attenuation and corrosion resistance (CR). SiC nanofibers, with their moderate dielectric constant and chemical inertness, are ideal multifunctional coating fillers. However, their single‐component nature limits microwave attenuation, resulting in low efficiency and narrow bandwidth. Incorporating corrosion‐resistant components and employing heterointerface engineering offers a promising strategy to enhance polarization loss and synergistically improve CR. In this study, SiC nanofibers synthesized via chemical vapor deposition are used as precursors; SiO_2_ interlayers and nitrogen‐doped carbon shells are sequentially introduced to form multilayered core–shell nanofibers. Abundant heterointerfaces and defects effectively regulate impedance matching and introduce multiple loss mechanisms, including conduction, interfacial, and defect‐induced dipole polarization. The prepared SiC@SiO_2_@C (SSC) nanofibers achieve a minimum reflection loss of −52.40 dB, a maximum effective absorption bandwidth of 7.68 GHz, and a maximum radar cross‐section reduction of 38.42 dB m^2^, demonstrating excellent MA properties. Moreover, SSC/polyvinylidene fluoride (PVDF) composite coatings exhibit superior CR performance, with significantly enhanced corrosion potential and reduced current density compared to pure metal and PVDF coatings. This study underscores the synergistic effect of heterointerface engineering in enhancing both MA and CR for harsh environments.

## Graphical abstract

The novel heterointerface engineering strategy integrates a SiO_2_ interlayer and amorphous carbon shell onto SiC nanofibers, enhancing microwave absorption and corrosion resistance.

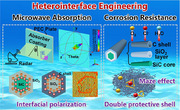

## 1 Introduction

As maritime defense and transportation become increasingly vital, equipment operating in marine environments must not only evade radar detection and tracking but also resist corrosion caused by high humidity and salt fog.^[^ ^1^, ^2^ ^]^ Therefore, developing multifunctional electromagnetic materials that integrate both efficient microwave absorption (MA) and outstanding corrosion resistance (CR) is essential for enhancing the survivability and operational stability of maritime equipment. Such materials can simultaneously suppress electromagnetic interference and protect underlying components from environmental degradation, thereby meeting the practical needs of next‐generation marine technologies. Silicon carbide (SiC) ceramics are widely recognized for their excellent CR and are frequently used as functional coating fillers^[^ ^3^ ^]^ or reinforcement phases^[^ ^4^ ^]^ in structural composites to maintain long‐term stability in corrosive environments. During operation, a compact SiO_2_ passivation layer spontaneously forms on the SiC surface, serving as an effective barrier against corrosive species such as Cl^−^.^[^ ^5^ ^]^ Moreover, the relatively low dielectric constant of SiC facilitates impedance matching with incident microwaves, making it a promising candidate for MA applications.^[^ ^6^ ^]^ Therefore, SiC is ideal for MA and CR applications. Despite these advantages, their relatively low dielectric losses and high filler loadings limit their applications in electromagnetic protection.^[^ ^7^ ^]^ Compared with SiC powder, fabricating 1D SiC nanofibers is advantageous because their anisotropic structure facilitates directional electron transport and network formation, thereby promoting multiple internal scattering and reflection of microwaves.^[^ ^8^ ^]^ This fibrous network is expected to reduce the filler content and enhance microwave dissipation.^[^ ^9^ ^]^

Nevertheless, the electromagnetic attenuation capacity of single‐component SiC nanofibers remains insufficient, leading to a low absorption efficiency and limited absorption bandwidth.^[^ ^10^ ^]^ To overcome this, various strategies have been proposed to enhance the performance of microwave absorbers, including magnetic‐dielectric synergy,^[^ ^11^ ^]^ interface engineering,^[^ ^12^, ^13^ ^]^ and mixed‐dimensional structures.^[^ ^14^ ^]^ Among these, heterointerface engineering has emerged as a simple yet effective approach for optimizing MA performance. By constructing heterogeneous core–shell structures, it significantly enhances interfacial polarization losses, thereby improving the overall attenuation capability of the material^[^ ^15^ ^]^ Heterointerfaces, composed of materials with different work functions, generate built‐in electric fields that promote directional charge migration and transition under alternating electromagnetic fields, thereby facilitating polarization energy dissipation. Wang et al.^[^ ^16^ ^]^ employed in situ chemical vapor deposition (CVD) to fabricate Fe_2_N@CNTs with heterointerfaces and encapsulation structures. The heterointerface improved dielectric loss, achieving a minimum reflection loss (RL_min_) of −54.55 dB at a thickness of 3.43 mm and an effective absorption bandwidth (EAB) of 5.52 GHz. This strategy confirmed the effectiveness of heterointerface construction in 1D materials, such as CNTs, for enhancing MA performance. However, further improvements in loss capacity are required to meet the current demands for broadband absorption.

Notably, SiC nanofibers have high aspect ratios and specific surface areas, providing abundant interfacial coupling sites when coated with an appropriate proportion of shells. The increased interfacial area enhances the energy consumption per unit volume of the system, thereby intensifying polarization losses and dissipating microwaves more effectively.^[^ ^17^ ^]^ Furthermore, the incorporation of corrosion‐resistant components into heterointerfaces offers a promising strategy for simultaneously improving the loss capacity of the SiC nanofibers and ensuring long‐term core protection. in situ oxidation of SiC to form SiC@SiO_2_ heterostructures has emerged as a widely adopted strategy in heterointerface engineering.^[^ ^18^ ^]^ Controlled oxidation expands the impedance‐matching area and introduces interfacial polarization while further thickening the protective oxide layer, preventing performance degradation caused by defects or damage in corrosive environments. Moreover, carbon coatings doped with heteroatoms such as nitrogen, sulfur, or phosphorus exhibit tunable electronic properties, rendering them highly suitable for use as encapsulating layers. These coatings not only enhance interfacial polarization losses but also protect the core from corrosion.^[^ ^19^ ^]^ Li et al.^[^ ^20^ ^]^ synthesized vanadium nitride@N‐doped carbon (VN‐F@NC) fibers via the nitrogen reduction of polydopamine‐coated (PDA)‐coated V_2_O_5_ precursor fibers, confirming that N‐doped carbon nanolayers effectively enhance both MA and CR properties. Similarly, Ge et al.^[^ ^21^ ^]^ fabricated N‐doped carbon‐encapsulated CoNi composites with different morphologies using metal–organic frameworks (MOFs) as templates. The rod‐like composite (CoNi/C‐r‐700), treated at 700 °C, exhibited an RL_min_ of −64.0 dB and an EAB of 4.8 GHz. The sample demonstrated excellent anti‐corrosion properties owing to its multilayer graphite shell. Therefore, constructing multilayered corrosion‐resistant interfaces (such as oxide and carbon layers) on SiC nanofibers not only enhances the loss characteristics of the material but also stabilizes its dielectric constant and MA performance under harsh conditions.

In this study, heterointerface engineering was applied to SiC nanofibers synthesized via CVD, in which in situ oxidation was first used to construct SiC@SiO_2_, followed by N‐doped carbon encapsulation to form multilayered core–shell SiC@SiO_2_@C (SSC) nanofibers. The phase composition, microstructure, MA, CR, and underlying mechanisms were systematically investigated. The results demonstrate that controlling the amount of carbon encapsulation effectively regulates impedance matching and strengthens polarization losses, leading to excellent MA performance with an ultrawide EAB at low filling ratios. Benefiting from the dual protection of the amorphous SiO_2_ interlayer and carbon shell, along with the physical barrier effect of the network structure against corrosive media, SSC materials exhibited excellent CR performance. This study provides valuable insights into the role of heterointerface engineering in high‐performance microwave‐absorbing materials for corrosion‐resistant applications.

## 2 Results and Discussion

### 2.1 Microstructure, Phase, and Chemical Composition of the Nanofibers

**Figure 1:**
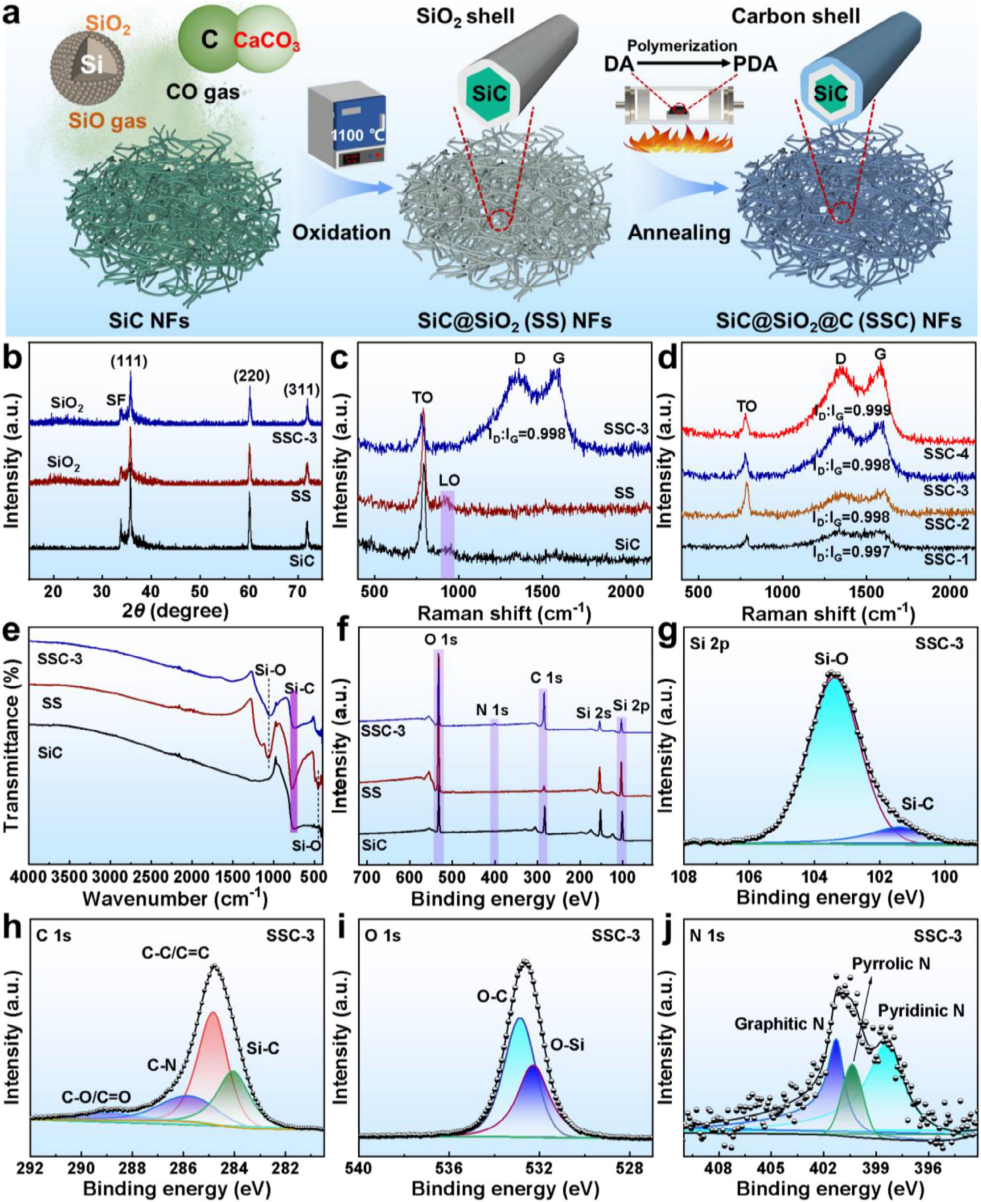
a) Schematic of the preparation process of SiC and SSC nanofibers. b) XRD patterns, c,d) Raman spectra, e) FTIR spectra, and f) XPS survey spectra of SiC, SS, and SSC. g) Si 2p, h) C 1s, i) O 1s, and j) N 1s high‐resolution XPS spectra of SSC‐3.

**Figure** **1** **a** illustrates the synthesis process of multi‐shelled SSC core–shell nanofibers. First, SiC nanofibers were synthesized using CVD, where a disproportionation reaction between Si and SiO_2_ produced SiO gas, whereas the decomposition of CaCO_3_ produced CO_2_ gas, which reacted with activated carbon to generate CO gas. Subsequently, SiO and CO gases facilitated the growth of SiC nanofibers on the surface of the graphite cover. These nanofibers then underwent in situ oxidation, followed by PDA coating in varying amounts and subsequent carbonization, resulting in the formation of SSC nanofibers. Figure 1b shows the XRD patterns of the samples. The main diffraction peaks correspond to the (111), (220), and (311) planes of 3C‐SiC (JCPDS No. 29–1129). Additionally, the weak peak at 33.6° is attributed to a stacking fault of the (111) plane.^[^ ^22^ ^]^ The broad peak at 2θ = 26° in the SS and SSC‐3 samples confirms the presence of the SiO_2_ phase, consistent with the partial oxidation of SiC nanofibers during in situ oxidation at 1100 °C. Raman spectra (Figure 1c) exhibit two characteristic SiC and SS peaks at 795 and 972 cm^−1^, corresponding to transverse optical (TO) and longitudinal optical (LO) modes of Si–C vibrations at the Γ point.^[^ ^23^ ^]^ Note that, owing to the amorphous structure of the carbon shell, the Raman intensity of the SSC‐series samples was significantly weaker than that of SiC. As shown in Figure 1d, the Raman spectra of the different carbon‐coated samples further reveal that the intensity ratio of the D and G peaks (*I*_D_:*I*_G_) increases with coating thickness. However, the change was relatively small, suggesting that the degree of graphitization of the SSC materials remained consistent at this carbonization temperature.

The Fourier transform infrared (FTIR) spectra (Figure 1e) show additional Si–O absorption bands at ≈1058 and 453 cm^−1^ in SS and SSC‐3 compared to SiC, whereas all three samples display a Si–C stretching vibration peak at ≈776 cm^−1^. As shown in Figure 1f, the X‐ray photoelectron spectroscopy (XPS) survey reveals that the C 1s peak of SSC‐3 is significantly enhanced, and an N 1s peak appears, confirming the presence of a N‐doped carbon layer, contributing to polarization loss. Figure 1g presents the Si 3d spectrum of SSC‐3, where two peaks at binding energies of 101.3 and 103.4 eV correspond to Si─C and Si─O bonds, respectively. The C 1s spectrum (Figure 1h) can be deconvoluted into four peaks: C─Si at 284.1 eV, C─C/C═C at 284.8 eV, C─N at 285.8 eV, and C–O/C═O at 288.9 eV. The O 1s spectrum (Figure 1i) shows two characteristic peaks at 532.2 and 532.8 eV, which correspond to the O─Si and O─C bonds, respectively. The presence of C─N bonds suggests the successful nitrogen doping of the carbon layer. To further confirm this, the N 1s spectrum, shown in Figure 1j, reveals three peaks at 398.4, 400.4, and 401.3 eV, corresponding to pyridinic N, pyrrolic N, and graphitic N, respectively.^[^ ^24^ ^]^ These results demonstrate the formation of N‐doped composites. Notably, nitrogen doping induces an uneven charge distribution between C and N atoms, leading to the formation of dipoles. Under an external electromagnetic field, these dipoles undergo orientation polarization, which facilitates the conversion of electromagnetic energy into other forms of energy.

**Figure 2:**
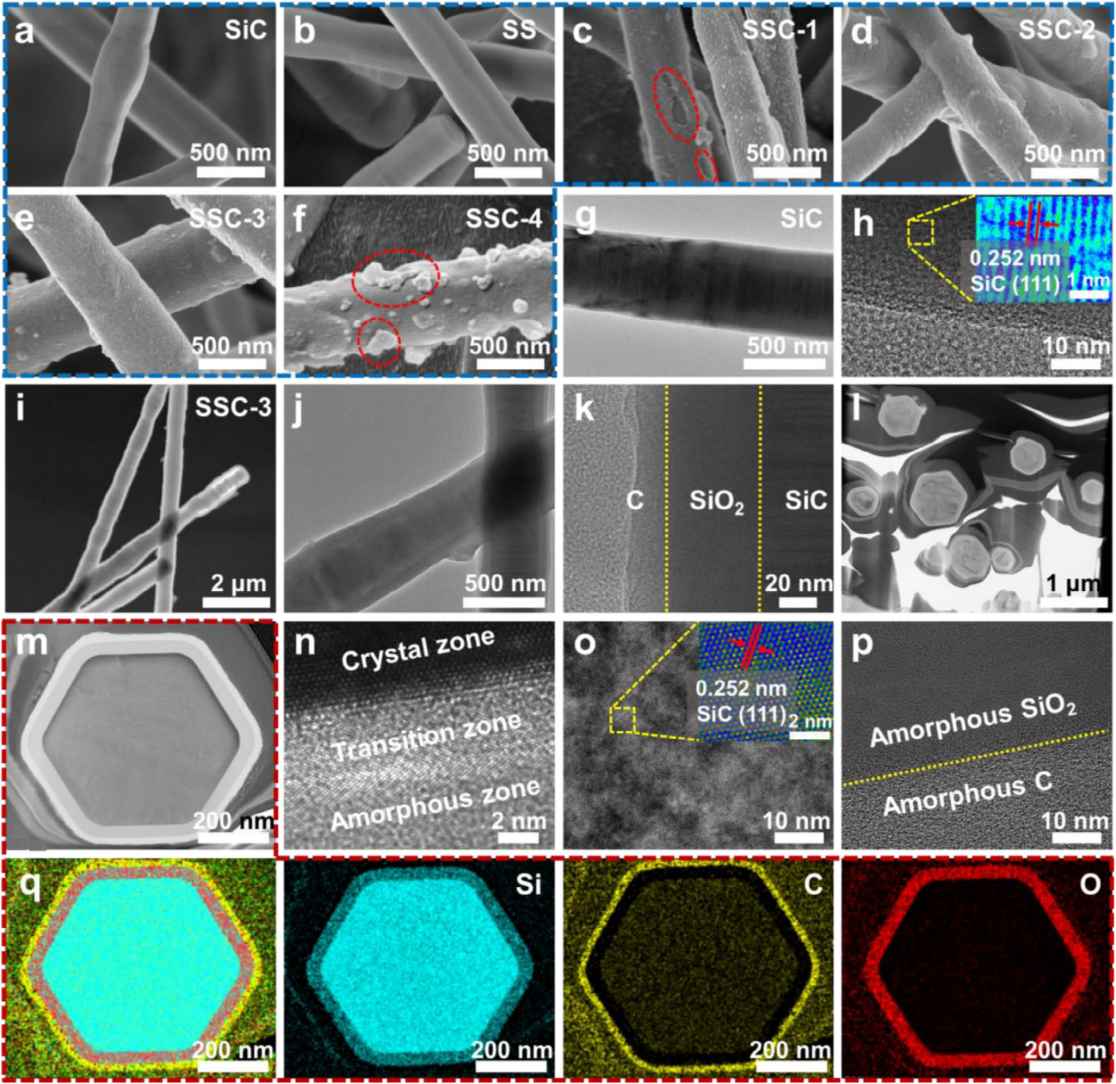
SEM images of a) SiC, b) SS, c) SSC‐1, d) SSC‐2, e) SSC‐3, and f) SSC‐4. g) TEM and h) HRTEM images of SiC. i,j) TEM images and k) HRTEM image of SSC‐3. Cross‐sectional microstructure of the SSC‐3 sliced by a focused ion beam (FIB): l,m) TEM images, n–p) HRTEM images, and m) EDS mapping.

The SEM morphologies of the fibrous materials were investigated, as shown in **Figures** **2**a–f and S1 (Supporting Information). Figure 2a and Figure S1a (Supporting Information) reveal that the as‐prepared SiC exhibits a prismatic morphology with a diameter of 150–400 nm, a smooth surface, and a length extending up to several hundred micrometers. After in situ oxidation (Figure 2b; Figure S1b, Supporting Information), the surface morphology remained nearly unchanged, with a slight increase in the nanofiber diameter. Figure 2c–f and Figure S1c–f (Supporting Information) present SEM images of the SSC nanofibers obtained by sequentially coating SS with PDA of varying thicknesses, followed by carbonization. A rough carbon layer enveloped the nanofiber surfaces. SSC‐1 exhibited sparse coating with surface gaps, whereas SSC‐3 showed uniform encapsulation. In SSC‐4, surface aggregation of carbonaceous particles became evident, indicating overcoating (Figure 2c–f), which helped us determine the optimal DA concentration.

Figure 2g–q and Figures S2–S6 (Supporting Information) present the transmission electron microscopy (TEM), high‐resolution transmission electron microscopy (HRTEM), and energy‐dispersive spectroscopy (EDS) mapping analyses of the SiC and SSC‐3 samples. Figure 2g, and Figures S2 and S3 (Supporting Information) confirm the formation of SiC, with homogeneously distributed Si and C. Furthermore, the HRTEM image (Figure 2h) shows a lattice spacing of 0.252 nm corresponding to the (111) plane of SiC.^[^ ^25^ ^]^ TEM analysis of SSC‐3 (Figure 2i,j) reveals its nanofibrous morphology. Furthermore, the HRTEM image (Figure 2k) identifies two distinct heterointerfaces, confirming the successful realization of this interface engineering strategy. Elemental distribution mapping (Figure S4, Supporting Information) further confirms that these phases correspond to the N‐doped carbon shell, intermediate SiO_2_ layer, and SiC core. FIB‐TEM characterization further elucidated the morphology of the SSC‐3 cells, as shown in Figure 2l and Figure S5 (Supporting Information). The cross‐sectional morphology of SSC exhibited a hexagonal structure, further verifying that the synthesized SiC nanofibers possessed a prismatic hexagonal geometry. Figure 2m and Figure S6a show two distinct heterointerfaces: SiC/SiO_2_ and SiO_2_/C, with a transition zone at the SiC/SiO_2_ interface where crystalline SiC gradually transformed into amorphous SiO_2_ (Figure 2n). The HRTEM analysis (Figure 2o) also reveals a lattice spacing of 0.252 nm, further confirming that the inner core consisted of crystalline SiC. Figure 2p shows amorphous SiO_2_ and C layers, which enhance the CR of SSC in seawater. Figure 2q and Figure S6b (Supporting Information) display the EDS mapping of the SSC cross section, providing a more intuitive visualization than Figure S4 (Supporting Information). This mapping further confirms the successful construction of multishell nanofibers through interfacial engineering, which holds promise for enhancing the microwave attenuation capability of SiC nanofibers.

### 2.2 MA Properties of the SSC Nanofibers

**Figure 3:**
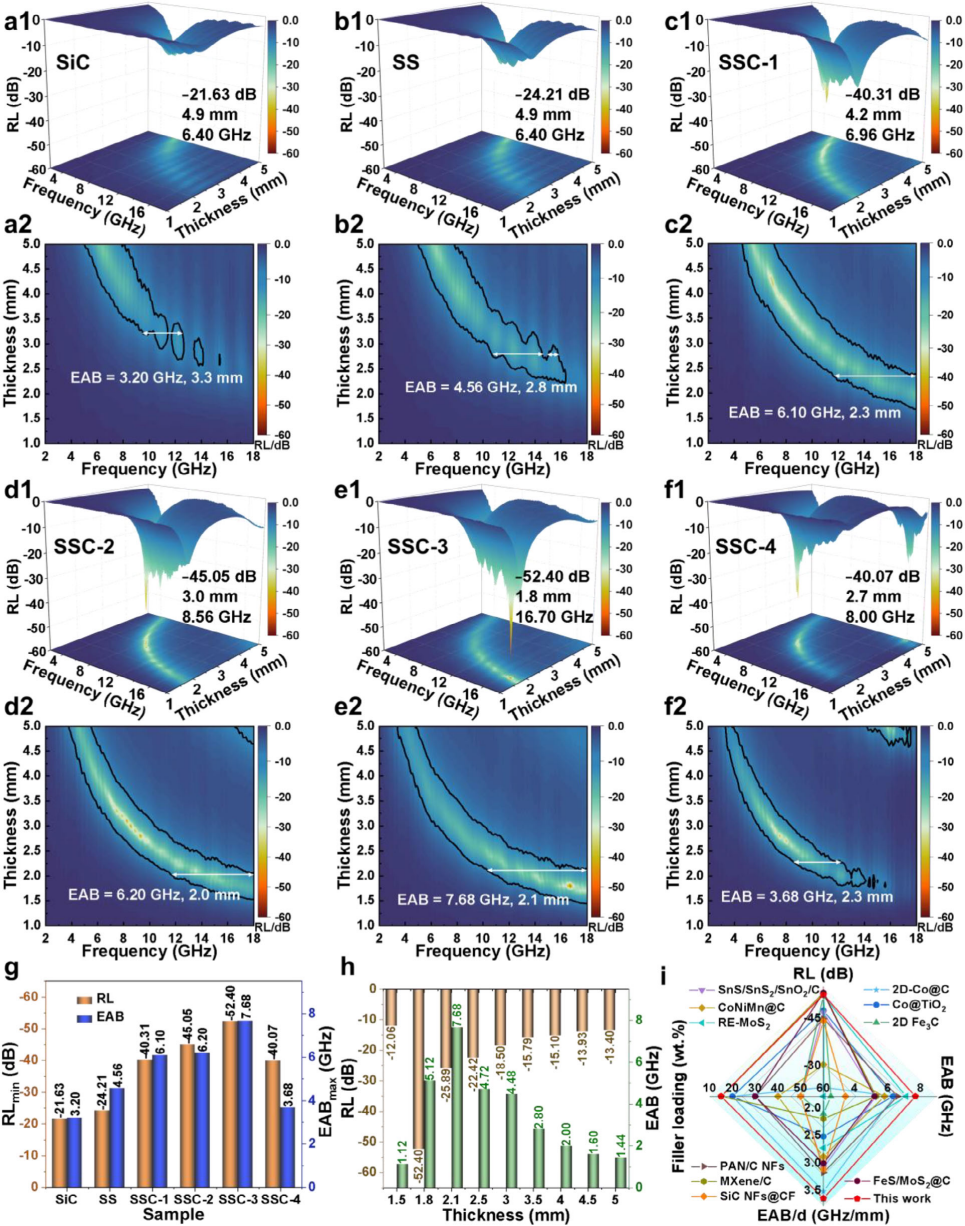
3D and 2D plots of reflection loss (RL) for a1,a2) SiC, b1,b2) SS, c1,c2) SSC‐1, d1,d2) SSC‐2, e1,e2) SSC‐3, and f1,f2) SSC‐4. g) RL_min_ and EAB_max_ for all samples. h) RL and EAB of SSC‐3 with varying thickness. i) Comparison of MA performance.

The influence of interfacial engineering on the electromagnetic properties of SiC nanofibers was investigated based on the transmission line theory by analyzing the complex electromagnetic parameters, including complex permittivity [ε_r_ = ε′−jε″] and permeability [μ_r_ = μ′−jμ″]. Here, the real parts (ε′, μ′) and imaginary parts (ε″, μ″) indicate the material's energy storage and attenuation capabilities from dielectric and magnetic perspectives, respectively.^[^ ^26^ ^]^ **Figure** **3** presents the MA performance of all nanofiber samples. As illustrated in Figure 3a–g, oxidation treatment slightly enhanced the RL_min_ of SiC, increasing from −21.63 to −24.21 dB. However, the EAB was broadened by 1.36 GHz, indicating that the SiC/SiO_2_ interface enhanced the absorption region of SiC. Following further carbon encapsulation, all SSC nanofiber samples exhibited RL_min_ values below −40 dB. Among them, SSC‐3 achieved an RL_min_ of −52.40 dB, corresponding to an absorption efficiency exceeding 99.999% at a matching thickness of only 1.8 mm. Additionally, the EAB values of SSC‐1, SSC‐2, and SSC‐3 increased beyond 6 GHz, classifying them as broadband microwave absorbers. Notably, SSC‐3 achieves an ultrawide EAB of 7.68 GHz, which is 2.4 times that of SiC and 1.7 times that of SS, demonstrating that the interfacial effect of SiO_2_/C is fully utilized under this carbon encapsulation condition. However, further increasing DA content reduces the EAB of SSC‐4 to 3.68 GHz (Figure 3f2), likely due to impedance mismatch leading to electromagnetic wave reflection, as discussed later. Figure 3h presents the variations in RL_min_ and EAB of SSC‐3 at different thicknesses, confirming its strong electromagnetic wave attenuation capability. To emphasize the advantages of heterointerface engineering, we comprehensively compared SSC with state‐of‐the‐art microwave absorbers, including carbon‐coated composites, MXene‐based materials, magnetic materials, and SiC‐based materials (Figure 3i; Table S1, Supporting Information).^[^ ^12^, ^23^, ^27^, ^28^, ^29^, ^30^, ^31^, ^32^, ^33^, ^34^ ^]^ The results reveal that the prepared fibrous material exhibited an excellent combination of low filler content, broad absorption bandwidth, and strong attenuation, making it a highly effective microwave absorber.

### 2.3 MA Mechanism of the SSC Nanofibers

To clarify the mechanism underlying the enhanced MA performance of the SSC materials, we first investigated their impedance‐matching characteristics (Figures S7 and S8, Supporting Information). As shown in Figure S7a1–c1, a2–c2 (Supporting Information), the absorption behavior of all samples follows the quarter‐wavelength (𝜆/4) theory, which is expressed by the following Equation:^[^ ^35^ ^]^

(1) $$t_{m}=\frac{n\lambda }{4}=\frac{nc}{\left(4f_{m}{\left(\left|\mu_{r}\left|\;\right|\varepsilon_{r}\right|\right)}^{\frac{1}{2}}\right)}\;,\left(n\;=1,3,5\text{…}\right)$$

where *t*_m_ and *f*_m_ represent the matching thickness and the corresponding frequency, respectively. When *t*_m_ and *f*_m_ satisfy Equation (1), a 180° phase difference between the incident and reflected waves leads to destructive interference. The simulated *t*_m_ values show good agreement with the experimental results, confirming that the quarter‐wavelength 𝜆/4 model effectively accounts for the regulation of RL values through adjustments in coating thickness. Notably, SSC‐3 effectively absorbed microwaves in the 3.36–18 GHz range at thicknesses of 1–5 mm, achieving a tunable EAB of up to 14.64 GHz. As shown in Figure S7a3–c3 (Supporting Information), the high‐loss RL_min_ values predominantly appear in regions where the impedance matching parameter |Z_in_/Z_0_| approaches 1, indicating minimal wave reflection. This suggests that dielectric constant tuning effectively modulates the impedance matching of SSC. Figure S8 (Supporting Information) further demonstrates that oxidation treatment broadens the impedance matching region, with |Z_in_/Z_0_| values falling between 0.8 and 1.2.^[^ ^36^ ^]^ As a result, the impedance matching area proportion increases from 16.06% for pristine SiC to 17.30% for SS. Subsequent carbon encapsulation further enlarges this proportion to 19.78% for SSC‐1, indicating that a thin carbon coating effectively improves impedance matching by facilitating greater microwave penetration and enhancing the potential for energy dissipation. However, with continued thickening of the carbon shell, the matching area proportion progressively declines, ultimately decreasing to 9.73% for SSC‐4, highlighting the detrimental effect of excessive encapsulation on impedance matching performance. This phenomenon highlights the intrinsic tradeoff between impedance matching and attenuation capacity in microwave‐absorbing materials.^[^ ^8^ ^]^ The interfacial engineering strategy, which integrates a conductive carbon layer with the dielectric SS matrix, shifts the impedance matching region to the mid‐to‐high frequency range at reduced thicknesses (Figure S7e, Supporting Information) while maintaining a matching area proportion of 10.80%. Simultaneously, it significantly enhances microwave attenuation in this frequency range, achieving a synergistic effect of strong loss and broadband absorption.

**Figure 4:**
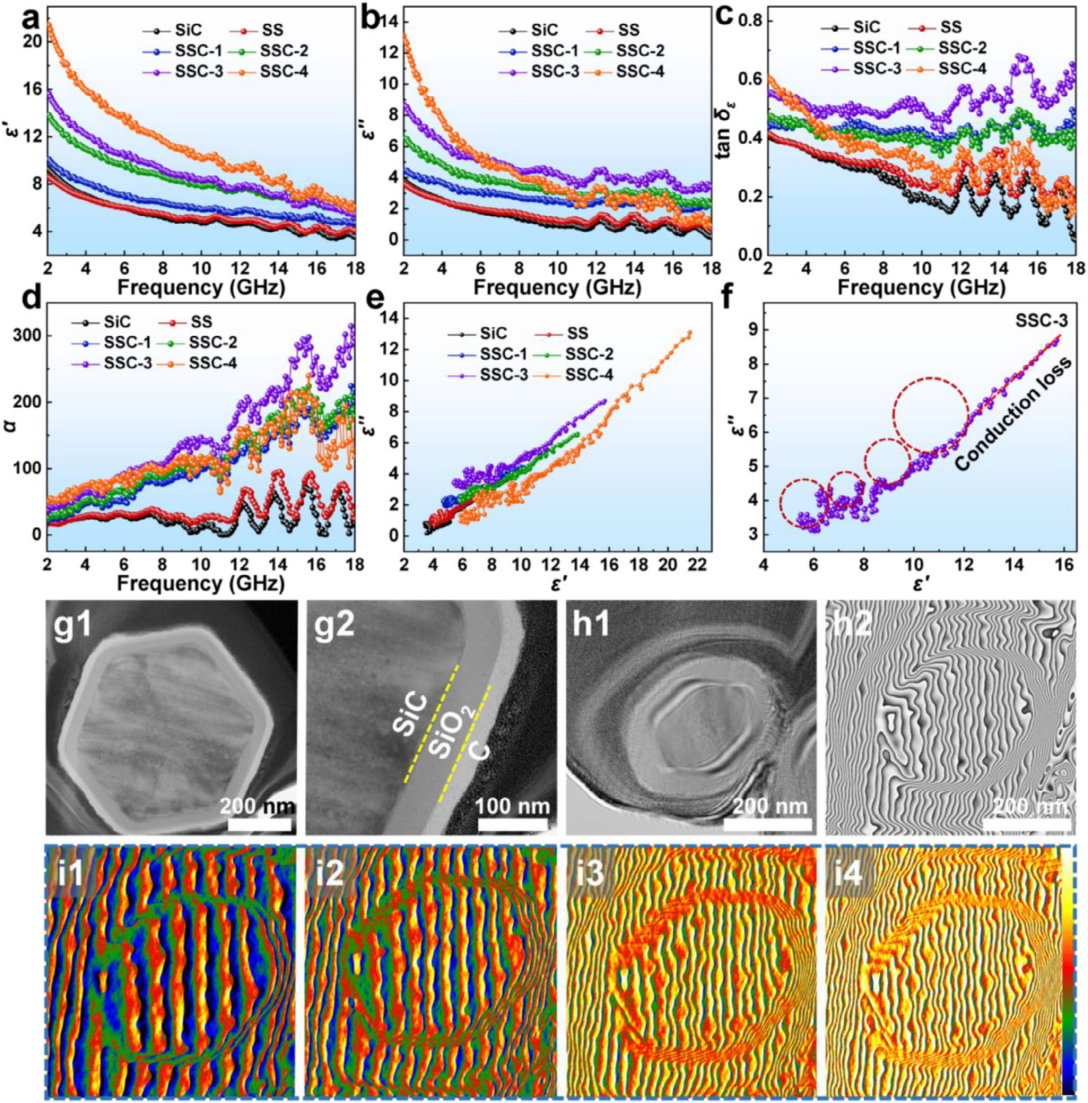
a) *ε*', b) *ε*'', c) dielectric tangent loss (tan *δ*_ε_), d) attenuation constant (*α*), and e) *ε*''–*ε*' curves of all samples. f) Cole–Cole semicircle curve of SSC‐3. g1,g2) TEM images, h1,h2) Off‐axis electron holograms, and i1–i4) Charge density image with further increase of electrical signal (light yellow as the color of the high charge density) of the SSC‐3 cross‐section.

Further investigation of the loss mechanisms of the SiC‐based nanofiber materials was conducted. As these materials do not contain magnetic components, their *μ*′ and *μ*″ values approach 1 and 0, respectively. **Figure** **4**a,b displays the frequency‐dependent real (*ε*′) and imaginary (*ε*″) components of the dielectric constant. The *ε*′ values exhibit a decreasing trend with increasing frequency, consistent with typical frequency dispersion behavior. In the low‐frequency region, the *ε*′ and *ε*″ values of SS are comparable to those of pristine SiC; however, in the mid‐to‐high frequency range, they surpass those of SiC, suggesting that the oxidation treatment enhances the dielectric loss capability of SiC in these regimes. This observation is further validated by the dielectric loss tangent (tan *δ*_ε_) curve in Figure 4c. As carbon encapsulation increases, the ε′ value of the materials gradually increases. Notably, SSC‐4 exhibits a maximum ε′ exceeding 20 within the 2–18 GHz range, making it highly susceptible to impedance mismatch. Additionally, SSC‐3 demonstrates the highest ε″ and tan δ_ε_ values in the 8–18 GHz range, exhibiting the most favorable dielectric loss characteristics. Furthermore, the attenuation constant (*α*) serves as a critical parameter for assessing a material's ability to dissipate microwave energy, expressed as follows:^[^ ^37^ ^]^

(2)

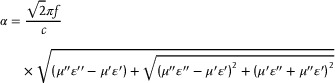




As shown in Figure 4d, SSC‐3 exhibits the highest α value, indicating that the nanofibers under this interfacial process possess the best incident wave attenuation rate. To further reveal the microwave loss mechanism of SiC‐based nanofiber materials, the *ε*″‐*ε*′ curves, such as the Cole–Cole semicircular curves, were plotted (Figure 4e). Based on the Debye polarization relaxation theory, the relationship between *ε*' and *ε*'' is governed by:^[^ ^38^ ^]^

(3) $$\varepsilon^{\prime }=\varepsilon_{\infty }+\frac{\varepsilon_{s}-\varepsilon_{\infty }}{1+{\left(2\pi f\right)}^{2}\tau^{2}},$$

(4) $$\varepsilon^{\prime \prime }=\frac{2\pi f\tau \left(\varepsilon_{s}-\varepsilon_{\infty }\right)}{1+{\left(2\pi f\right)}^{2}\tau^{2}}\;$$

(5) $${\left(\varepsilon^{\prime }-\frac{\varepsilon_{s}-\varepsilon_{\infty }}{2}\right)}^{2}+{\left(\varepsilon^{\prime \prime }\right)}^{2}={\left(\frac{\varepsilon_{s}-\varepsilon_{\infty }}{2}\right)}^{2}\;$$

where *ε*_s_ and *ε*_∞_ are the static dielectric constant and high‐frequency limiting dielectric constant, respectively, and *τ* and *ω* (*ω* = 2*πf*) are the polarization relaxation time and angular frequency, respectively. Equation (5) indicates that a polarization relaxation process produces semicircular curves in *ε*′–*ε*″ plots, with the semicircle representing a Debye relaxation process. As illustrated in Figure 4f and Figure S9 (Supporting Information), the Cole–Cole plots of the prepared nanofibers exhibit distortions, transitioning into a straight line in the latter half of the curve, suggesting a pronounced conduction loss effect.^[^ ^39^ ^]^ A comparison of the number of Cole‐Cole semicircles for all samples indicates that SSC‐3 undergoes more polarization relaxation processes (Figure S9f, Supporting Information), which can be attributed to the dipole polarization of defect‐induced charge formation and the interfacial polarization between the multicomponent inhomogeneous interfaces in the composite.

The preceding analysis confirms that SSC materials consist of a SiC core, an in situ‐oxidized SiO_2_ interlayer, and an outermost N‐doped carbon shell. These heterogeneous interfaces induce strong interfacial polarization, effectively dissipating microwave energy. Notably, the SiO_2_ layer is positioned between SiC and C, both of which exhibit higher electrical conductivity than SiO_2_, creating two regions resembling “capacitor‐like” structures (Figure 4g1,g2). Using off‐axis electron holography, the heterogeneous interface region of SSC‐3 was successfully visualized (Figure 4h1,h2), providing an effective approach for analyzing the charge density distribution and dielectric loss characteristics.^[^ ^40^ ^]^ Lorentz TEM observations revealed a well‐defined SiC–SiO_2_–C structure in SSC‐3 cross‐sections. Under an alternating electromagnetic field, free carriers accumulate at interfaces and defects during transport. As depicted in Figure 4i1, the uneven distribution of carriers at the SiC/SiO_2_ and SiO_2_/C interfaces formed space‐charge polarization zones that displace the geometric centers of the overlapping lattices and generate interfacial polarization.^[^ ^41^ ^]^ In addition, the enhanced electromagnetic signal strength promoted increased charge accumulation within the polarized regions, thereby intensifying interfacial polarization, as illustrated in Figure 4i1–i4. Ultimately, this technique successfully reveals how interfacial engineering enhances polarization loss, thereby improving the MA performance.

**Figure 5:**
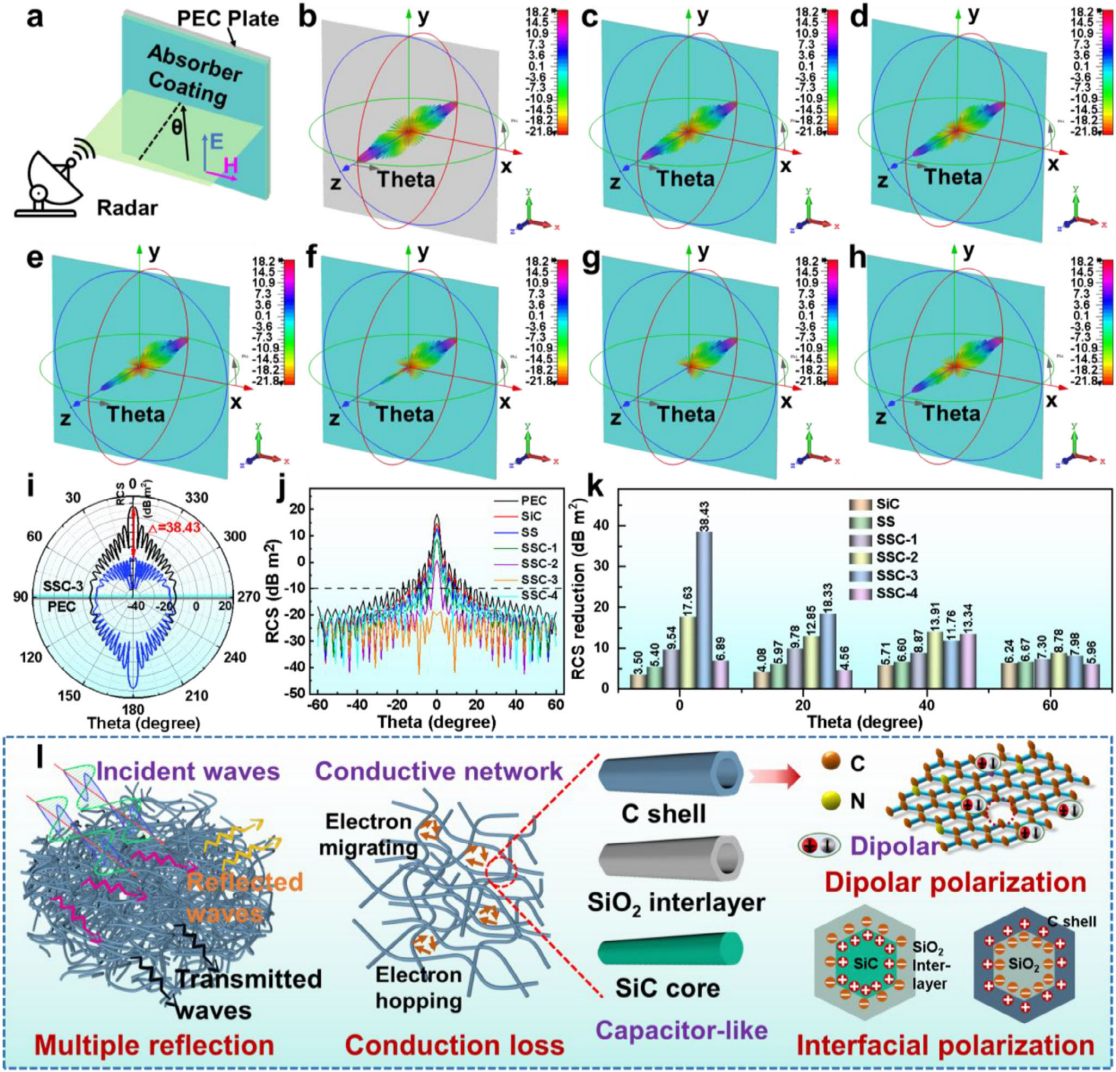
a) Schematic of the absorber‐coated metal backplane. 3D RCS simulation results for b) PEC plate, PEC plate coated with c) SiC, d) SS, e) SSC‐1, f) SSC‐2, g) SSC‐3, and h) SSC‐4. i) RCS in polar coordinates for the PEC plate covered with SSC‐3. j) RCS values of all the samples. k) RCS reduction values of these samples at selected scanning angles. l) Schematic of the MA mechanism of SSC.

**Figure** **5**a illustrates the working principles of the CST Studio Suite, which align with the metal‐backed absorber model in transmission line theory to simulate the radar cross section (RCS) reduction for targets under far‐field conditions.^[^ ^42^ ^]^ Figure 5b–h presents the RCS results for perfect electric conductor (PEC) plates with and without SiC‐based coatings, demonstrating that the uncoated PEC plate exhibits the strongest scattering signals, whereas all nanofiber‐coated samples significantly reduce 3D radar wave scattering compared to pure PEC. Figure 5i,j further indicates that SSC‐3 exhibits superior stealth performance, achieving the lowest RCS values, consistently below −15 dB m^2^ within an angular range of −60°–60°, underscoring the advantages of interfacial design. Additionally, RCS reduction, a critical parameter in stealth technology, is defined as the difference between the RCS of an uncoated PEC plate and that of the same plate coated with the material. As shown in Figure 5k, SSC‐3 achieves the highest RCS reduction of 38.43 dB m^2^ at θ = 10°, demonstrating its potential as an advanced electromagnetic shielding material for stealth applications.

Based on the above analysis, the MA mechanism of SSC materials is summarized in Figure 5l. First, the in situ‐grown oxide layer on the SiC nanofiber surface and the encapsulating carbon shell effectively modulate the impedance matching of the material. Second, unlike conductors with free electrons, the electrons in pristine SiC nanofibers are in a bound state, and the SiO_2_ interlayer is electrically insulating. However, following carbon shell encapsulation, the SiC@SiO_2_@C nanofibers acquire a continuous one‐dimensional conductive carbon layer, establishing an interconnected network that facilitates additional electron transport through both migration and hopping conduction mechanisms. Under an external electromagnetic field, electrons radially flow along the SSC nanofibers, propagating rapidly through the network, facilitating faster electromagnetic energy dissipation. Moreover, electron hopping between the nanofiber networks further enhances conduction loss, while multiple reflections and scattering within the network contribute to effective energy dissipation. Meanwhile, nitrogen atoms have higher electronegativity than carbon, and when incorporated into the carbon framework, they form C─N bonds (e.g., pyridinic‐N, pyrrolic‐N, and graphitic‐N), which introduce asymmetric electron distributions. These features facilitate the formation of responsive electric dipoles under alternating electromagnetic fields, resulting in enhanced dipolar polarization. In addition, nitrogen doping induces lattice distortion, vacancies, and disruptions in the *π*‐conjugated system within the carbon matrix, which can lead to charge redistribution and localized polarization, thereby increasing polarization loss. Most importantly, the high aspect ratio and large specific surface area of 1D SiC nanofibers provide numerous interfacial coupling sites, further increasing interfacial surface area. The SiO_2_ interlayer and carbon shell, introduced through heterointerface engineering, exhibit distinct work functions, resulting in an asymmetric charge distribution across the heterojunctions and the formation of space charge regions, also known as built‐in electric fields. Under an alternating electromagnetic field, these Si/SiO_2_ and SiO_2_/C heterointerfaces promote directional charge migration and transition, enhancing interfacial polarization. Moreover, the increased interfacial surface of the 1D structure further intensifies the energy dissipation per unit volume, maximizing polarization loss and efficiently dissipating microwaves.

### 2.4 CR Properties and Mechanism of SSC Nanofibers

**Figure 6:**
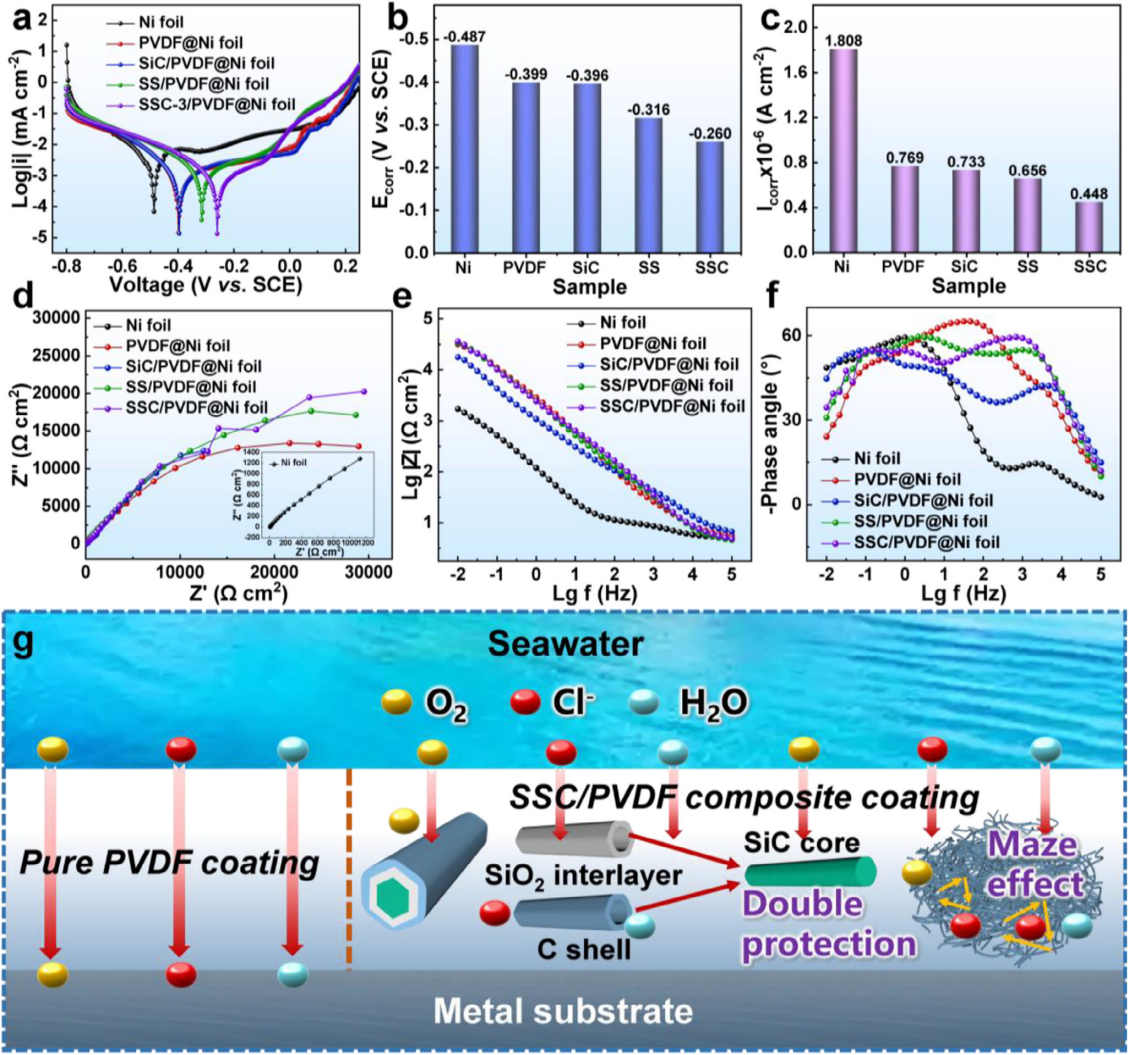
Electrochemical analysis of Ni foil, pure PVDF coating, and nanofibers/PVDF composite coatings in a 3.5 wt.% NaCl solution. a) Tafel curves, b) Corrosion potential (*E*_corr_), c) Corrosion current density (*I*_corr_), d) Nyquist plots, e) Bode plots, and f) Phase angle plots. g) Schematic of the corrosion protection mechanism.

Microwave absorbers for marine environments must exhibit excellent MA and CR performance. The corrosion resistance of coatings is commonly assessed via electrochemical measurements employing a three‐electrode configuration, with a 3.5 wt.% NaCl aqueous solution serving as a simulated seawater electrolyte. **Figure** **6**a presents the Tafel curves of various samples, where bare Ni foil exhibits the lowest corrosion potential (*E*_corr_), followed by the pure polyvinylidene fluoride (PVDF) coating. Upon incorporating the nanofibers, all composite coatings demonstrate higher corrosion potentials than the pure Ni or PVDF coatings, with the SSC/PVDF composite achieving the highest value (Figure 6b). Furthermore, the corrosion current density (*I*_corr_) of the SSC/PVDF composite, obtained through curve fitting, was the lowest among all samples (Figure 6c). A higher *E*_corr_ or lower *I*_corr_ indicates greater CR, confirming that the interfacial engineering design enhances the CR performance of SSC/PVDF compared with that of SiC/PVDF, primarily because of the presence of the SiO_2_ layer and encapsulating carbon shell.^[^ ^43^ ^]^ The Nyquist plots of bare Ni and the coated samples (Figure 6d) demonstrate that the semicircle radius of bare Ni is markedly smaller than that of the coated counterparts, indicating a pronounced vulnerability to corrosion. All composite coatings exhibit larger impedance arc radii than the pure epoxy coating, demonstrating improved corrosion protection for bare Ni, with SSC/PVDF offering the most effective barrier. In the Bode plot, the impedance modulus at 0.01 Hz (|Z|_0.01 Hz_) is a key indicator of antiresistance. As shown in Figure 6e, all the coatings exhibit significantly higher |Z|_0.01 Hz_ values than bare Ni, with the SSC/PVDF composite slightly outperforming the pure PVDF coating, further validating its superior anticorrosion performance.^[^ ^44^ ^]^ All coatings displayed higher phase angles than bare Ni across the frequency range of 10^−2^–10^5^ Hz (Figure 6f), indicative of pronounced capacitive behavior that effectively impedes corrosive species penetration. Furthermore, the phase‐angle peak of bare Ni is located closer to the low‐frequency region (10^−2^–10^0^ Hz) compared with the coated samples, which corresponds to the corrosion response of the metallic substrate, confirming that corrosion occurs immediately upon immersion.^[^ ^45^ ^]^ These findings indicate that the incorporation of the SSC nanofibers significantly enhanced the CR of the PVDF coatings.

Figure 6g illustrates the corrosion protection mechanism of the composite coatings. Typically, in pure PVDF coatings, defects and micropores compromise compactness, allowing corrosive species (Cl^−^, H_2_O, O_2_) to penetrate easily, accelerating coating degradation. Consequently, the pure PVDF coating provided only limited protection to the metallic substrate. In contrast, the SSC‐reinforced composite coating exhibited substantially improved CR performance. On the one hand, interfacial engineering introduces two inert layers (SiO_2_ and C) into the SiC nanofibers, forming a dual protective shell. Meanwhile, the encapsulated carbon shell, which undergoes high‐temperature carbonization, attains a high degree of graphitization, significantly inhibiting electrochemical corrosion reactions. On the other hand, the interconnected 3D network of nanofibers creates complex and tortuous diffusion pathways, which not only enhance the structural compactness of the coating but also effectively extend the penetration path of corrosive agents, thereby improving the overall corrosion resistance. This results in a “maze effect,” which effectively enhances the physical shielding capability of the coating.^[^ ^46^ ^]^ In summary, the interfacial engineering design of SSC materials strengthens the anticorrosion capability of PVDF coatings, both chemically and physically, making them highly suitable for practical applications in complex electromagnetic environments.

## 3 Conclusion

This study employed heterointerface engineering to fabricate multilayered SSC nanofibers for multifunctional MA and CR coatings. The synergistic enhancement mechanisms arising from multiple interfaces on the MA and anticorrosion properties of SiC nanofibers were systematically investigated. The formation of abundant heterointerfaces (SiC/SiO_2_ and SiO_2_/C) induced abundant interfacial polarization relaxation processes, thereby enhancing dielectric loss. As a result, SSC‐3 achieved an RL_min_ of −52.40 dB, a maximum RCS reduction of 38.42 dB m^2^, and an EAB_max_ of 7.68 GHz, which is 2.4 times that of pure SiC, demonstrating its potential as a high‐performance absorbing coating filler. In addition, the SSC/PVDF composite coating exhibited superior CR performance. The amorphous SiO_2_ interlayer and carbon shell confer dual protective barriers, while the interconnected nanofiber network prolongs diffusion pathways, establishing a “maze effect” that significantly enhances physical shielding. This study deepens the understanding of high‐performance MA materials, demonstrates the synergistic enhancement of MA and CR through heterointerface engineering, and offers a promising strategy for the development of multifunctional electromagnetic materials suitable for complex and harsh environments.

## 4 Experimental Section

### Raw Materials

Activated carbon (analytical grade) and calcium carbonate (CaCO_3_, analytical grade) were purchased from Sigma–Aldrich (USA). Silicon dioxide (SiO_2_, analytical grade, 40–80 mesh), Si powder (99.99%, 40–200 mesh), and N‐methyl‐2‐pyrrolidone (NMP, >99.0%) were purchased from Aladdin Ltd. (Shanghai, China). Tris‐HCl buffer (pH 8.8), 3.5 wt.% NaCl solution, polyvinylidene fluoride (PVDF, Mw ≈534,000), and dopamine hydrochloride (DA, 99.8%) were purchased from Macklin Biochemical Co., Ltd. (Shanghai, China).​

### Synthesis of SiC Nanofibers

Silicon carbide nanofibers were prepared according to a previously reported method.^[^ ^47^ ^]^ Briefly, a mixture of activated carbon and CaCO_3_ in a 1:1 molar ratio served as the carbon source, whereas a mixture of SiO_2_ and Si powder in a molar ratio of 1:1 served as the silicon source. The mixture was ball milled at 200 rpm for 3 h to ensure uniform mixing. Subsequently, the carbon and Si sources were placed on opposite sides of a rectangular graphite crucible. The crucible was then placed in a high‐temperature tube furnace, heated under an argon atmosphere at a rate of 5 °C min^−1^ to 1500 °C, and maintained at that temperature for 5 h. After cooling to room temperature, SiC nanofibers were obtained. During the high‐temperature reduction process, intermediate gaseous products SiO and CO were generated from the reaction between the carbon and silicon sources, forming a highly volatile and diffusive reducing atmosphere at 1500 °C. These gases migrated along the gas flow direction and were preferentially deposited and reacted on the surface of the graphite‐covered region, leading to the formation of SiC nanofibers. Following cooling of the graphite crucible to ambient temperature, the graphite cover bearing the deposited SiC nanofibers was transferred to a muffle furnace and subjected to oxidation at 700 °C for 2 h, enabling efficient exfoliation of the SiC nanofibers.

### Synthesis of SiC@SiO_2_@C Nanofibers

The SiC nanofibers were evenly spread in an alumina crucible and heated in a muffle furnace under an air atmosphere at a rate of 5 °C min^−1^ to 1100 °C, where they were held for 10 min to form SiC@SiO_2_ (denoted as SS), as reported in our previous study.^[^ ^47^ ^]^ SS (100 mg) was dispersed in Tris‐HCl buffer solution and stirred for 20 min. Various amounts of DA were added, and the mixture was stirred at room temperature for 12 h, allowing DA to undergo oxidative self‐polymerization in the alkaline solution and adhere to the SS surface, forming SS@PDA. The thicknesses of the PDA shells were controlled by adjusting the amount of DA. Finally, SS@PDA was heated in a tube furnace under an argon atmosphere at a rate of 5 °C min^−1^ to 800 °C, held for 2 h, and carbonized to yield SiC@SiO₂@C nanofibers, denoted as SSC‐x, where x corresponds to DA amounts of 50, 100, 150, and 200 mg, labeled as SSC‐1, SSC‐2, SSC‐3, and SSC‐4, respectively.

### Characterization

The phase composition of the materials was determined by X‐ray diffraction (XRD, Bruker D8‐Discover, Cu K_α_ radiation, operated at 40 kV). Raman spectra of the materials were obtained using a confocal Raman microscope (alpha300 Access, WITec GmbH). The chemical structures were analyzed using Fourier‐transform infrared spectroscopy (FTIR, Nicolet iS10, Thermo Fisher Scientific). The microstructures of the SSC samples were observed using field‐emission scanning electron microscopy (FESEM; FEI Nova 450). TEM, HRTEM) EDS mapping, and off‐axis electron holography were performed using field‐emission TEM (JEM‐2100F). The surface chemical composition was determined using XPS (Thermo ESCALAB 250XI). Scanning transmission electron microscopy (STEM, FEI Themis Z) was used to identify the atomic structures of the sliced samples using a focused ion beam (FIB, FEI Helios NanoLab 600i).

The electromagnetic parameters were measured by dispersing the samples uniformly in paraffin and pressing them into ring‐shaped specimens (filler ratio of 15 wt.%, inner diameter 7.00 mm, outer diameter 3.04 mm, thickness ≈2.00 mm) using molds. Paraffin was selected as the matrix primarily because its electromagnetic parameters are similar to those of air, and it exhibits negligible electromagnetic loss. This minimizes interference from the matrix on the intrinsic microwave absorption performance of the material, allowing for an accurate evaluation of the filler's electromagnetic response behavior. A vector network analyzer (Agilent N5234A) was used to measure the complex permittivity and permeability of the samples. The RL of the material was calculated based on the transmission line theory using the following formulas:^[^ ^48^ ^]^

(6) $$\mathrm{RL}\;=20\mathrm{lg}\left|\frac{Z_{\mathrm{in}}-Z_{0}}{Z_{\mathrm{in}}+Z_{0}}\right|$$

(7) $$Z_{\mathrm{in}}=Z_{0}\;\sqrt{\mu_{r}/\varepsilon_{r}}\tanh\left[j\left(\frac{2\pi \mathrm{fd}}{c}\right)\sqrt{\mu_{r}\varepsilon_{r}}\right]$$

where *Z*_in_, *Z*_0_, *d*, *f*, and *c* represent the input impedance of the absorber, impedance of free space, thickness of the absorber, frequency, and speed of light, respectively. An RL value of less than −10 dB indicates that more than 90% of the electromagnetic wave energy is absorbed. Therefore, the frequency range with RL less than −10 dB was termed the effective absorption bandwidth (EAB).^[^ ^49^ ^]^

CST Studio Suite 2020 was used to simulate the radar cross‐section (RCS) of the microwave absorber under real far‐field response conditions. A metal‐backed model was used, consisting of a double‐layer square sample (20 × 20 cm^2^), with an upper absorbing layer (1.8 mm thick) and a lower perfect electric conductor (1.0 mm thick) plate. The sample/PEC was placed on the *x–y* plane, and linearly polarized electromagnetic waves were incident from the positive *z*‐axis to the negative *z*‐axis with electric‐field polarization along the *x*‐axis. Open boundary conditions were applied in all directions, and the field‐monitoring frequency was set to 16.7 GHz. The scattering direction was determined using the spherical coordinates Theta and Phi. The RCS value was given by:^[^ ^50^ ^]^

(8) $$\delta \left(\mathrm{dB}m^{2}\right)=10\mathrm{lg}{\left(\left(4\pi S/\lambda^{2}\right)\left|E_{s}/E_{i}\right|\right)}^{2}$$

where *S*, *λ*, *E*_s_, and *E*_i_ represent the area of the target simulation model, wavelength of the electromagnetic wave, scattered wave's electric field intensity, and incident wave's electric field intensity, respectively.

Electrochemical tests were conducted using a CHI660E electrochemical workstation (Chenhua, Shanghai, China) in 3.5 wt.% NaCl solution (simulated seawater) to obtain Tafel polarization curves and electrochemical impedance spectroscopy (EIS), with Tafel scans conducted at 0.01 mV s^−1^ over a voltage range of −0.8–0.4 V. This solution is widely recognized as a standard system for simulating chloride‐induced corrosion in marine environments and can effectively reflect the material's corrosion behavior under actual service conditions. EIS measurements were performed over a frequency range of 0.01–10^5^ Hz. The CR performance of the material was evaluated using a three‐electrode system with a platinum counter electrode and a saturated calomel electrode as the reference electrode. The SiC‐based absorber was uniformly mixed with PVDF in a 1:1 mass ratio, and an appropriate amount of NMP solution was added. Afterward, the mixture was ball‐milled into a uniform slurry, which was then coated onto the surface of the Ni foil using the doctor blade method. The coated samples were vacuum dried at 50 °C for 6 h before being punched into discs with a diameter of 16 mm using a stamping machine to serve as working electrodes. PVDF was chosen as the coating matrix due to its excellent chemical stability, corrosion resistance, and film‐forming ability, effectively protecting the absorber in salt spray environments and reflecting its real‐world performance.

## Conflict of Interest

The authors declare no conflict of interest.

## Supporting information



Supporting Information

## Data Availability

The data that support the findings of this study are available from the corresponding author upon reasonable request.

## References

[1] X. Xu , S. Shi , Y. Tang , G. Wang , M. Zhou , G. Zhao , X. Zhou , S. Lin , F. Meng , Adv. Sci. 2021, 8, 2002658. 10.1002/advs.202002658 PMC7927622 33717840

[2] H. Lv , Y. Yao , S. Li , G. Wu , B. Zhao , X. Zhou , R. L. Dupont , U. I. Kara , Y. Zhou , S. Xi , B. Liu , R. Che , J. Zhang , H. Xu , S. Adera , R. Wu , X. Wang , Nat. Commun. 2023, 14, 1982. 37031210 10.1038/s41467-023-37436-6 PMC10082851

[3] H. Guo , C. Yang , H. Sun , N. Xiang , C. Wang , Surf Interfaces 2024, 46, 104057.

[4] H. Qian , Z. Xu , S. Chen , Y. Liu , D. Yan , Surf Coat Tech 2022, 434, 128172.

[5] S. Yuan , K. Li , Y. Sun , C. Cong , Y. Liu , D. Lin , L. Pei , Y. Zhu , H. Wang , Chem. Eng. J. 2023, 472, 144881.

[6] Z. Jin , X. Feng , Y. Hou , H. Zhu , L. Wang , Chem. Eng. J. 2024, 498, 155110.

[7] Z. Wang , J. Liu , H. Hao , Q. Jing , S. Yan , J. Guo , Z. Wang , Carbon 2024, 217.

[8] Z. Wu , H. W. Cheng , C. Jin , B. Yang , C. Xu , K. Pei , H. Zhang , Z. Yang , R. Che , Adv. Mater. 2022, 34, 2107538. 10.1002/adma.202107538 34755916

[9] L. Wu , G. Wang , S. Shi , X. Liu , J. Liu , J. Zhao , G. Wang , Adv. Sci. 2023, 10, 2304218. 10.1002/advs.202304218 PMC10625052 37721442

[10] L. Song , F. Zhang , Y. Chen , L. Guan , Y. Zhu , M. Chen , H. Wang , B. R. Putra , R. Zhang , B. Fan , Nano‐Micro Lett. 2022, 14, 152. 10.1007/s40820-022-00905-6 PMC9334492 35900619

[11] J. Cheng , H. Zhang , M. Ning , H. Raza , D. Zhang , G. Zheng , Q. Zheng , R. Che , Adv. Funct. Mater. 2022, 32, 2200123.

[12] Y. Shen , Z. Ma , F. Yan , C. Zhu , X. Zhang , Y. Chen , Adv. Funct. Mater. 2025, 35, 2423947.

[13] F. Hu , S. Xie , F. Wu , J. Liu , P. Zhang , J. Ding , B. Fan , W. Zheng , L. Cai , Z. Sun , J. Mater. Chem. A 2024, 12, 33939.

[14] Q. Liang , M. He , B. Zhan , H. Guo , X. Qi , Y. Qu , Y. Zhang , W. Zhong , J. Gu , Nano‐Micro Lett. 2025, 17, 167. 10.1007/s40820-024-01626-8 PMC11865380 40009269

[15] Z. Ma , K. Yang , D. Li , H. Liu , S. Hui , Y. Jiang , S. Li , Y. Li , W. Yang , H. Wu , Y. Hou , Adv. Mater. 2024, 36, 2314233 10.1002/adma.202314233 38380795

[16] J. Wang , Z. Miao , K. Gao , Z. Li , X. Zhang , C. Yang , S. Iqbal , G. Xiang , A. Cui , L. Liu , C. Sun , H. Wu , J. Y. Yang , Adv. Funct. Mater. 2024, 34, 2408696.

[17] X. Li , X. Wang , M. Li , W. Zhu , H. Luo , X. Lu , H. Xu , J. Xue , F. Ye , H. Wu , X. Fan , Adv. Funct. Mater. 2024, 2407217.

[18] Z. Wang , Y. Hou , H. Hao , Y. Shuai , Z. Wang , Carbon 2023, 211, 118092.

[19] Y. Bi , M. Ma , Z. Liao , Z. Tong , Y. Chen , R. Wang , Y. Ma , G. Wu , J. Colloid Interface Sci. 2022, 605, 483. 34340035 10.1016/j.jcis.2021.07.050

[20] R. Li , J. Lu , C. Li , Y. Cui , D. Lv , Y. Chen , Y. Wei , H. Wei , B. Liang , J. Bu , Colloid Surface A 2024, 686, 133420.

[21] J. Ge , Y. Cui , J. Qian , L. Liu , F. Meng , F. Wang , J. Mater. Sci. Technol. 2022, 102, 24.

[22] L. Song , C. Wu , Q. Zhi , F. Zhang , B. Song , L. Guan , Y. Chen , H. Wang , R. Zhang , B. Fan , Chem. Eng. J. 2023, 467, 143518.

[23] W. Geng , Y. Liu , H. Lei , L. Song , H. Wang , G. Shao , Y. Zhu , R. Zhang , Z. Min , B. Fan , Chem. Eng. J. 2024, 499, 155785.

[24] Z. Gao , Z. Ma , D. Lan , Z. Zhao , L. Zhang , H. Wu , Y. Hou , Adv. Funct. Mater. 2022, 32, 2112294.

[25] C. Wang , X. Chen , Z. Wang , J. Bai , J. Tang , Y. She , Z. Huang , Y. Yang , J. Adv. Ceram. 2024, 13, 1212.

[26] X. Zeng , X. Jiang , Y. Ning , F. Hu , B. Fan , J. Adv. Ceram. 2023, 12, 1562.

[27] J. Wen , S. Hui , Q. Chang , G. Chen , L. Zhang , X. Fan , K. Tao , H. Wu , Adv. Funct. Mater. 2024, 34, 2410447.

[28] J. Xiao , B. Zhan , M. He , X. Qi , X. Gong , J. L. Yang , Y. Qu , J. Ding , W. Zhong , J. Gu , Adv. Funct. Mater. 2024, 2316722.

[29] Z. Gao , A. Iqbal , T. Hassan , S. Hui , H. Wu , C. M. Koo , Adv. Mater. 2024, 36, 2311411. 10.1002/adma.202311411 38288859

[30] Z. Guo , D. Lan , Z. Jia , Z. Gao , X. Shi , M. He , H. Guo , G. Wu , P. Yin , Nano‐Micro Lett. 2025, 17, 23. 10.1007/s40820-024-01527-w PMC11436513 39331208

[31] M. Huang , B. Li , Y. Qian , L. Wang , H. Zhang , C. Yang , L. Rao , G. Zhou , C. Liang , R. Che , Nano‐Micro Lett. 2024, 16, 245. 10.1007/s40820-024-01416-2 PMC11245463 38995472

[32] F. Wu , P. Hu , F. Hu , Z. Tian , J. Tang , P. Zhang , L. Pan , M. W. Barsoum , L. Cai , Z. Sun , Nano‐Micro Lett. 2023, 15, 194. 10.1007/s40820-023-01158-7 PMC10412520 37556089

[33] Y. Liu , J. Zhou , C. Li , H. Zhang , Y. Wang , Y. Yan , L. Duan , Z. Cheng , Y. Ma , Z. Yao , Nat. Commun. 2025, 16, 202. 39747194 10.1038/s41467-024-55776-9 PMC11696289

[34] R. Zhao , T. Gao , Y. Li , Z. Sun , Z. Zhang , L. Ji , C. Hu , X. Liu , Z. Zhang , X. Zhang , G. Qin , Nat. Commun. 2024, 15, 1497. 38374257 10.1038/s41467-024-45815-w PMC10876570

[35] F. Hu , P. Zhang , F. Wu , Z. Tian , H. Tang , B. Fan , R. Zhang , W. Sun , L. Cai , Z. M. Sun , J. Materiomics 2024, 10, 531.

[36] Y. Hou , Z. Sheng , C. Fu , J. Kong , X. Zhang , Nat. Commun. 2022, 13, 1227. 35264594 10.1038/s41467-022-28906-4 PMC8907192

[37] X. Wang , Y. Yuan , X. Sun , R. Qiang , Y. Xu , Y. Ma , E. Zhang , Y. Li , Small 2024, 20, 2311657. 10.1002/smll.202311657 38461547

[38] S. Hui , X. Zhou , L. Zhang , H. Wu , Adv. Sci. 2024, 11, 2307649. 10.1002/advs.202307649 PMC10853738 38044282

[39] F. Hu , H. Tang , F. Wu , P. Ding , P. Zhang , W. Sun , L. Cai , B. Fan , R. Zhang , Z. Sun , Small Methods 2024, 8, 2301476. 10.1002/smtd.202301476 38183383

[40] H. Niu , X. Tu , S. Zhang , Y. Li , H. Wang , G. Shao , R. Zhang , H. Li , B. Zhao , B. Fan , Chem. Eng. J. 2022, 446, 137260.

[41] B. Zhao , Z. Yan , Y. Du , L. Rao , G. Chen , Y. Wu , L. Yang , J. Zhang , L. Wu , D. W. Zhang , R. Che , Adv. Mater. 2023, 35, 2210243. 10.1002/adma.202210243 36606342

[42] W. Gu , S. J. H. Ong , Y. Shen , W. Guo , Y. Fang , G. Ji , Z. J. Xu , Adv. Sci. 2022, 9, 2204165. 10.1002/advs.202204165 PMC9762302 36285685

[43] S. Li , T. Xie , L. Ma , Z. Lei , N. Huang , H. Song , Y. Feng , B. Li , Y. Cui , L. Liu , W. Liu , B. Zhao , J. Zhang , R. Che , S. Ma , Z. Zhang , Carbon 2023, 213, 118302.

[44] S. Li , L. Ma , Z. Lei , A. Hua , A. Zhang , Y. Song , F. Liu , D. Geng , W. Liu , S. Ma , Z. Zhang , Carbon 2022, 186, 520.

[45] C. Zheng , M. Ning , Z. Zou , G. Lv , Q. Wu , J. Hou , Q. Man , R. W. Li , Small 2023, 19, 2208211. 10.1002/smll.202208211 37078912

[46] L. Duan , J. Zhou , J. Tao , Y. Liu , Y. Yan , Y. Wang , X. Yang , X. Tao , Z. Yao , H. Huang , P. Liu , Y. Ma , Compos Part B‐Eng. 2024, 287, 111882.

[47] L. Song , L. Wang , Y. Chen , H. Wu , B. Song , N. Wang , L. Guan , H. Wang , R. Zhang , Y. Zhu , Y. Xia , B. Fan , Small 2024, 20, 2407563. 10.1002/smll.202407563 39420747

[48] F. Wu , F. Hu , P. Hu , P. Zhang , B. Fan , H. Kong , W. Zheng , L. Cai , Z. Sun , Carbon 2024, 230, 119644.

[49] Z. Wang , F. Zhang , N. Wang , W. Li , Y. Chen , H. Wang , R. Zhang , Y. Zhu , B. Fan , J. Adv. Ceram. 2024, 13, 699.

[50] F. Hu , P. Ding , F. Wu , P. Zhang , W. Zheng , W. Sun , R. Zhang , L. Cai , B. Fan , Z. Sun , Carbon Energy 2024, 6, 638.

