# Supplementary material for: Heterointerface‐Engineered SiC@SiO2@C Nanofibers for Simultaneous Microwave Absorption and Corrosion Resistance
Source: Adv Sci (Weinh). 2025 Jul 31;12(37):e09071. doi: 10.1002/advs.202509071 (PMC12499453; doi:10.1002/advs.202509071)
Supplement: Supplementary file 1 — Supporting Information [file ADVS-12-e09071-s001.docx]

**Heterointerface-engineered SiC@SiO_2_@C nanofibers for simultaneous microwave absorption and corrosion resistance**

Limeng Song^1, 3^, Feiyue Hu^2*^, Yongqiang Chen^3^, Li Guan^1^, Peigen Zhang^2^^[[1]](#footnote-1)^, Linan Wang^4^, ZhengMing Sun^2^, Yanqiu Zhu^5^, Hailong Wang^3^, Renchao Che^6*^, Bingbing Fan^3, 5*^, and Rui Zhang^1, 3*^

1 School of Materials Science and Engineering, Zhengzhou University of Aeronautics, Zhengzhou 450015, P. R. China

2 State Key Laboratory of Engineering Materials for Major Infrastructure, School of Materials Science and Engineering, Southeast University, Nanjing 211189, P. R. China

3 School of Materials Science and Engineering, Zhengzhou University, Zhengzhou 450001, P. R. China

4 Institute of Advanced Ceramics, Henan Academy of Sciences, Zhengzhou 450046, P. R. China

5 Department of Engineering, Faculty of Environment, Science and Economy, University of Exeter, Exeter EX4 4QF, United Kingdom

6 Laboratory of Advanced Materials, Shanghai Key Lab of Molecular Catalysis and Innovative Materials, Academy for Engineering & Technology, Fudan University, Shanghai 200438, P. R. China


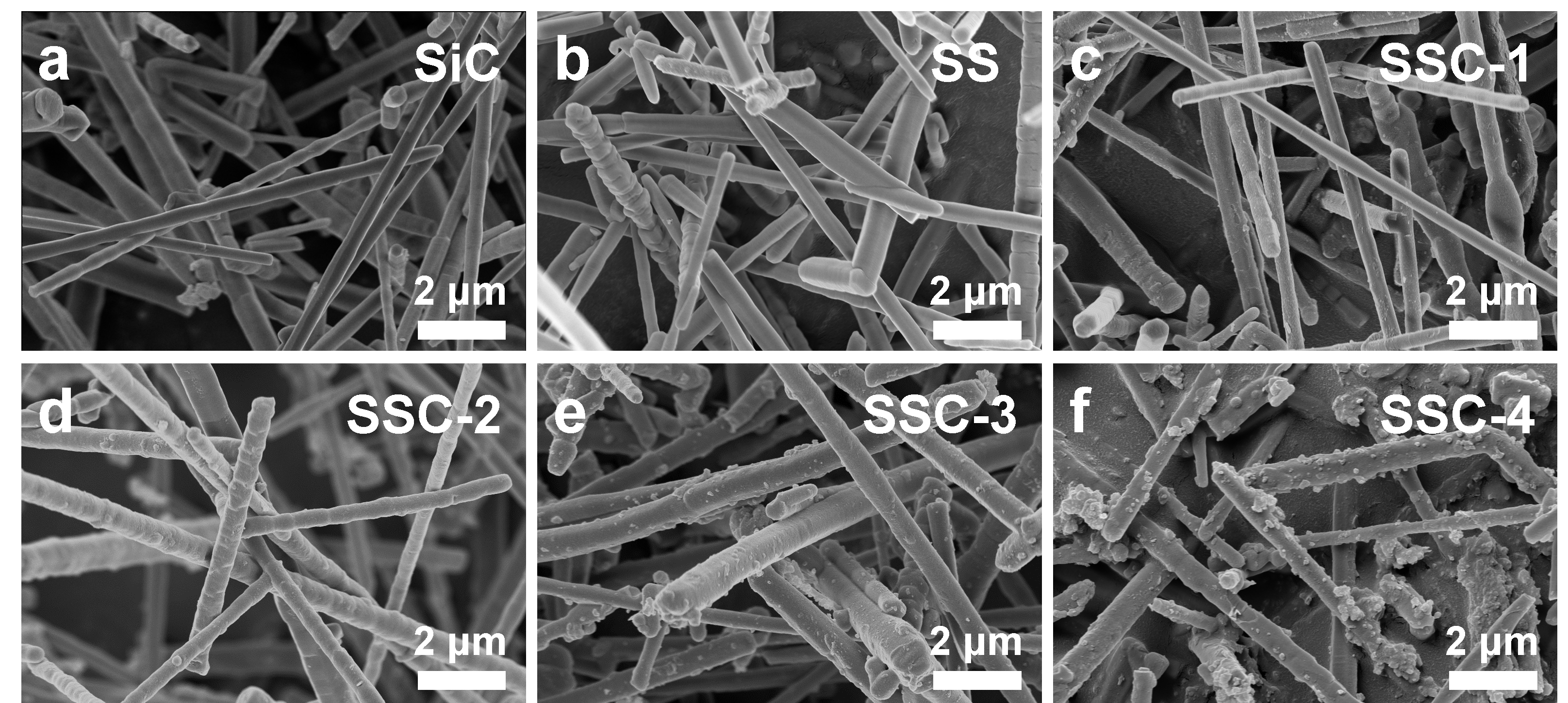


Figure S1. SEM images of a) SiC, b) SS, c) SSC-1, d) SSC-2, e) SSC-3, and f) SSC-4.


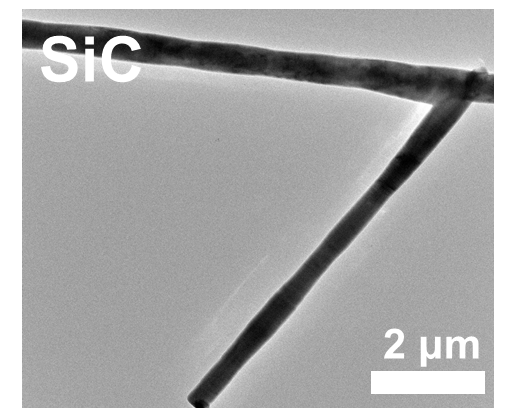


Figure S2. TEM image of SiC.


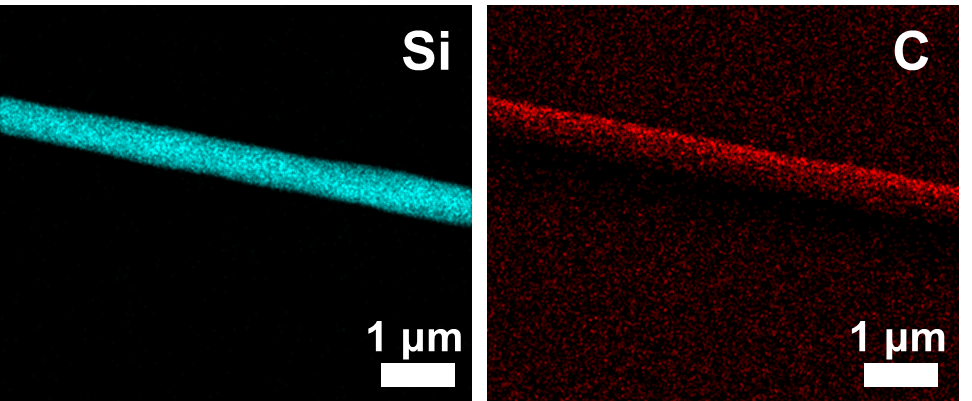


Figure S3. EDS mapping of SiC.


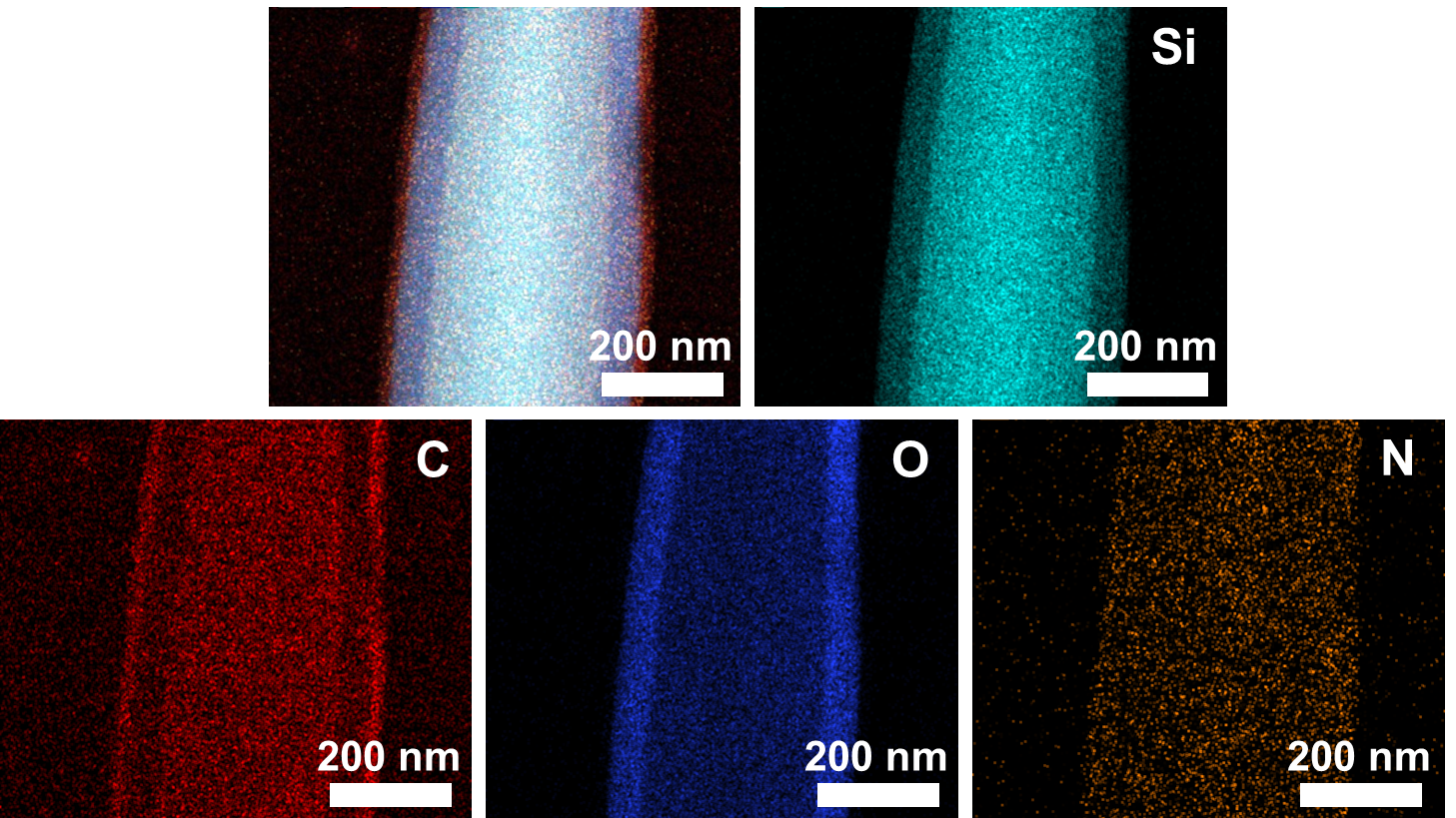


Figure S4. EDS mapping of SSC-3.


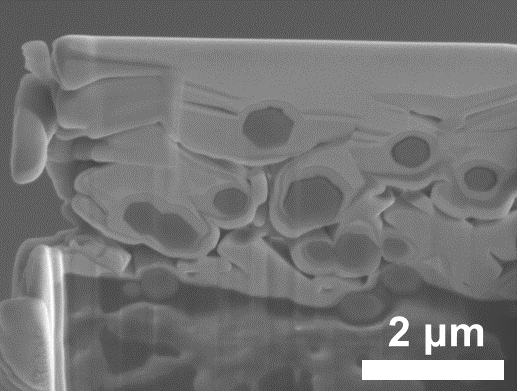


Figure S5. Cross-sectional microstructure of the SSC-3 sliced by a FIB: SEM image.


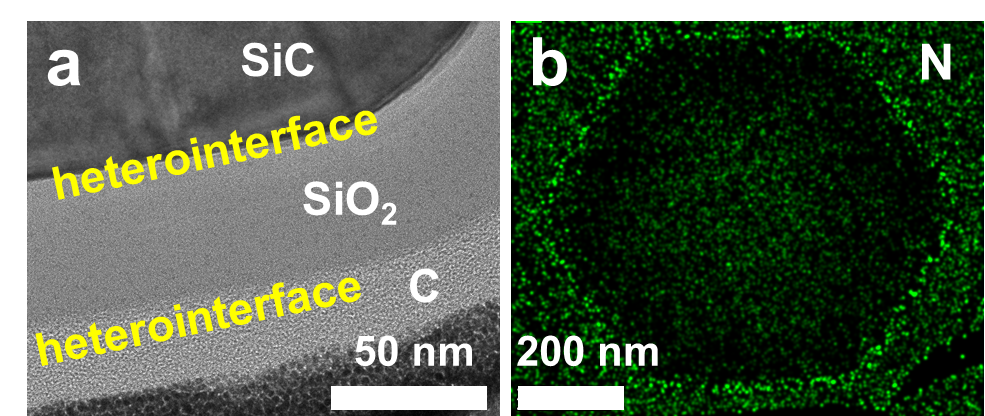


Figure S6. a) TEM image and b) EDS mapping (N element) of the cross section of SSC-3.


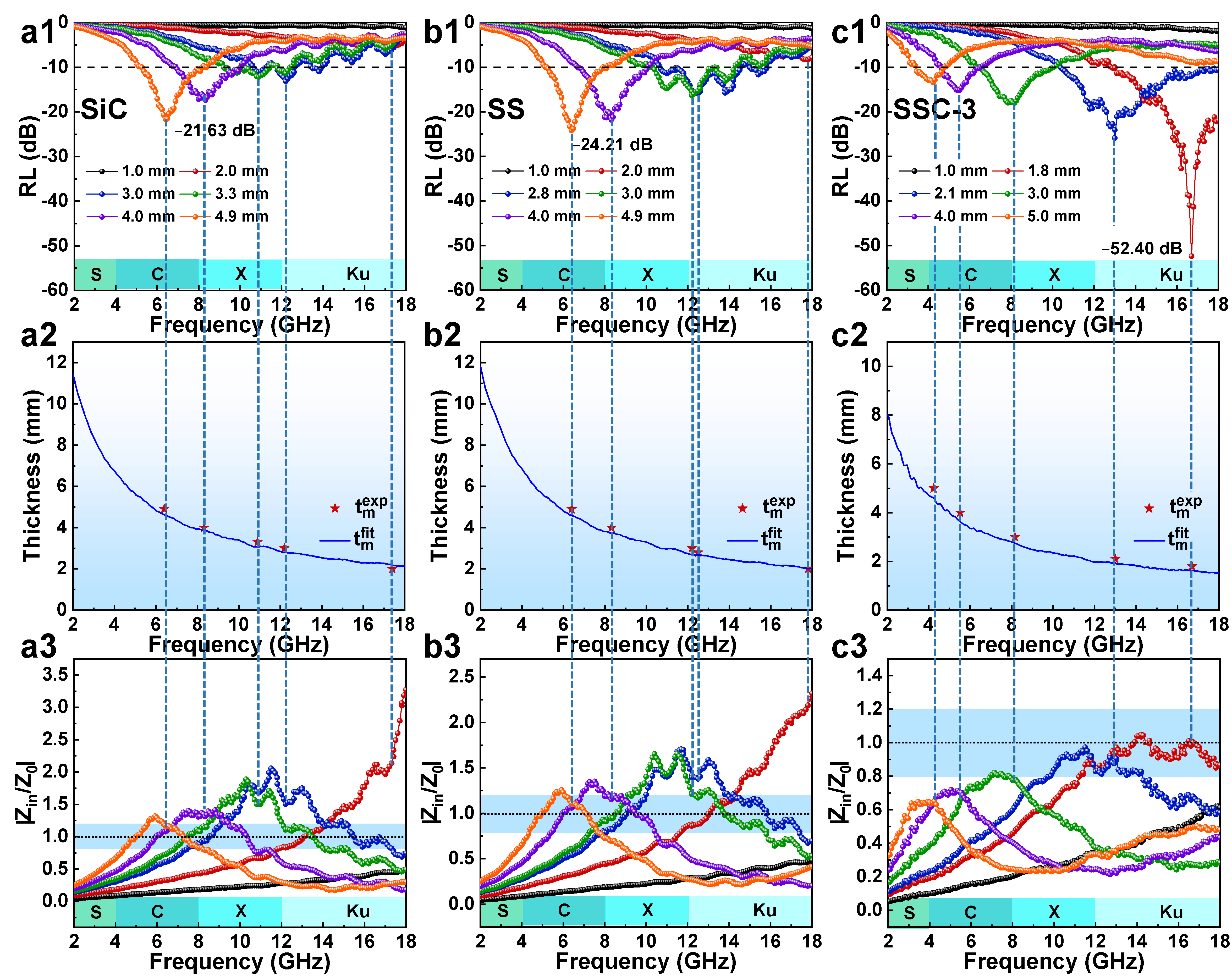


Figure S7. 2D plots of the RL, the relationship between thickness and peak frequency (quarter-wavelength mode), and impedance matching rate (|Z_in_/Z_0_|) at different thickness of a) SiC, b) SS, and c) SSC-3, respectively.


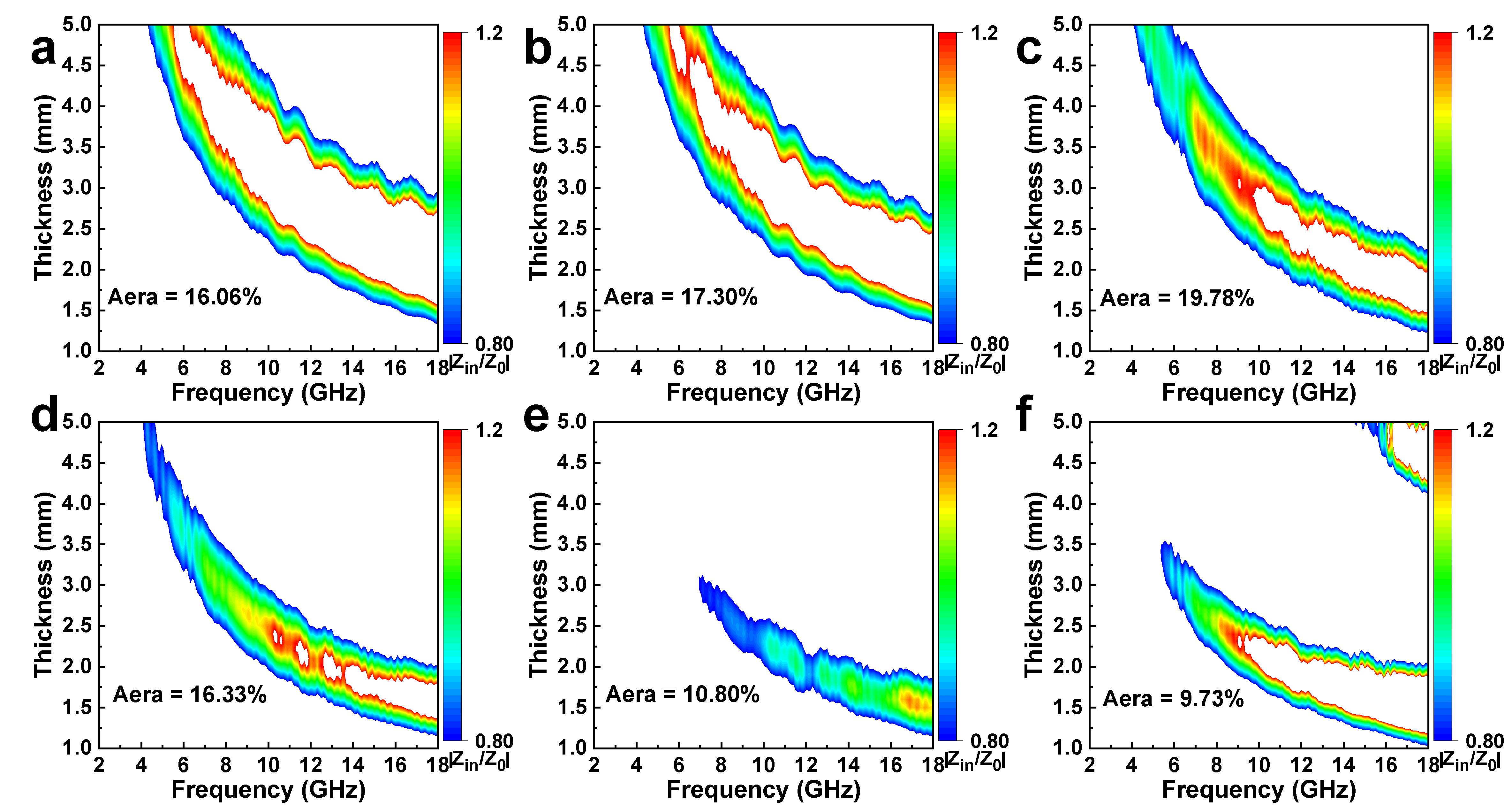


Figure S8. 2D representations of |Z_in_/Z_0_| at different thickness of a) SiC, b) SS, c) SSC-1, d) SSC-2, e) SSC-3, and f) SSC-4.


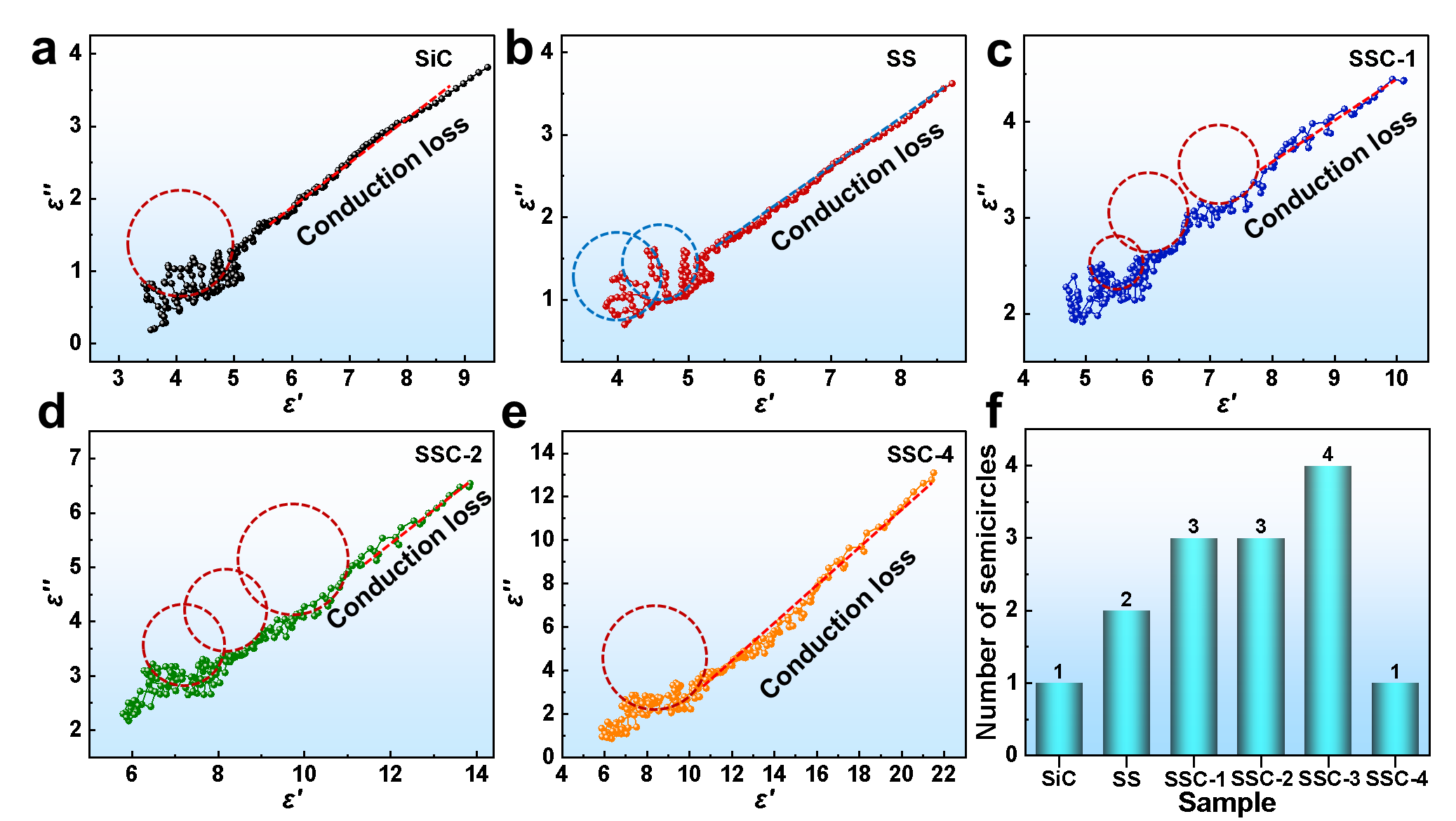


Figure S9. Cole-Cole semicircle curves of a) SiC, b) SS, c) SSC-1, d) SSC-2, and e) SSC-4. f) Number of semicircles of all samples.

**Table S1.** **MA properties of comparative absorbers.**

| Materials | Filler (wt.%) | RL_min_ (dB) | d_1_ (mm) | EAB (GHz) | d_2_ (mm) | EAB/d_2_ (GHz/mm) | Ref. |
| --- | --- | --- | --- | --- | --- | --- | --- |
| 2D-Co@C | 20 | -22.87 | 1.82 | 6.41 | 1.82 | 3.53 | [1] |
| Co@TiO_2_ | 20 | -47.35 | 2.5 | 6.32 | 2.5 | 2.53 | [2] |
| 2D Fe_3_C | 50 | -52.09 | 2.90 | 2.55 | 1.20 | 2.12 | [3] |
| SnS/SnS_2_/SnO_2_/CF | 50 | -46.74 | 2.0 | 5.28 | 1.7 | 3.10 | [4] |
| CoNiMn@C | 40 | -30.1 | 2.0 | 5.8 | 2.0 | 2.9 | [5] |
| RE-MoS_2_ | 60 | -52.02 | 2.6 | 7.12 | 2.6 | 2.74 | [6] |
| PAN/C NFs | 30 | -44.73 | 1.76 | 6.60 | 2.07 | 3.19 | [7] |
| MXene/C | 15 | -53.02 | 3.8 | 5.3 | 2.4 | 2.21 | [8] |
| SiC NFs@CF | 50 | -44.04 | 1.2 | 3.44 | 1.1 | 3.13 | [9] |
| FeS/MoS_2_@NC | 30 | -53.01 | 1.63 | 5.20 | 1.72 | 3.02 | [10] |
| **SSC-3** | **15** | **-52.40** | **1.8** | **7.68** | **2.1** | **3.66** | **Here** |

**Reference**

[1] Y. Liu, J. Zhou, C. Li, H. Zhang, Y. Wang, Y. Yan, L. Duan, Z. Cheng, Y. Ma, Z. Yao, *Nat Commun* **2025**, *16*, 202.

[2] Z. Gao, A. Iqbal, T. Hassan, S. Hui, H. Wu, C. M. Koo, *Adv Mater* **2024**, *36*, 2311411.

[3] R. Zhao, T. Gao, Y. Li, Z. Sun, Z. Zhang, L. Ji, C. Hu, X. Liu, Z. Zhang, X. Zhang, G. Qin, *Nat Commun* **2024**, *15*, 1497.

[4] Z. Guo, D. Lan, Z. Jia, Z. Gao, X. Shi, M. He, H. Guo, G. Wu, P. Yin, *Nano-Micro Lett* **2025**, *17*, 23.

[5] M. Huang, B. Li, Y. Qian, L. Wang, H. Zhang, C. Yang, L. Rao, G. Zhou, C. Liang, R. Che, *Nano-Micro Lett* **2024**, *16*, 245.

[6] J. Wen, S. Hui, Q. Chang, G. Chen, L. Zhang, X. Fan, K. Tao, H. Wu, *Adv Funct Mater* **2024**, *34*, 2410447.

[7] J. Xiao, B. Zhan, M. He, X. Qi, X. Gong, J. L. Yang, Y. Qu, J. Ding, W. Zhong, J. Gu, *Adv Funct Mater* **2024**, 2316722.

[8] F. Wu, P. Hu, F. Hu, Z. Tian, J. Tang, P. Zhang, L. Pan, M. W. Barsoum, L. Cai, Z. Sun, *Nano-Micro Lett* **2023**, *15*, 194.

[9] W. Geng, Y. Liu, H. Lei, L. Song, H. Wang, G. Shao, Y. Zhu, R. Zhang, Z. Min, B. Fan, *Chem Eng J* **2024**, *499*, 155785.

[10] Y. Shen, Z. Ma, F. Yan, C. Zhu, X. Zhang, Y. Chen, *Adv Funct Mater* **2025**, 2423947.

1. Corresponding authors. E-mail: hufeiyue@seu.edu.cn (F. Hu), zhpeigen@seu.edu.cn (P. Zhang), rcche@fudan.edu.cn (R. Che), fanbingbing@zzu.edu.cn (B. Fan), zhangray@zzu.edu.cn (R. Zhang). [↑](#footnote-ref-1)
